# TRP Channels Interactome as a Novel Therapeutic Target in Breast Cancer

**DOI:** 10.3389/fonc.2021.621614

**Published:** 2021-06-10

**Authors:** María Paz Saldías, Diego Maureira, Octavio Orellana-Serradell, Ian Silva, Boris Lavanderos, Pablo Cruz, Camila Torres, Mónica Cáceres, Oscar Cerda

**Affiliations:** ^1^Program of Cellular and Molecular Biology, Institute of Biomedical Sciences (ICBM), Faculty of Medicine, Universidad de Chile, Santiago, Chile; ^2^Millennium Nucleus of Ion Channels-Associated Diseases (MiNICAD), Santiago, Chile; ^3^The Wound Repair, Treatment, and Health (WoRTH) Initiative, Santiago, Chile

**Keywords:** TRP channels, protein–protein interactions, calcium signaling, breast cancer, interactomics

## Abstract

Breast cancer is one of the most frequent cancer types worldwide and the first cause of cancer-related deaths in women. Although significant therapeutic advances have been achieved with drugs such as tamoxifen and trastuzumab, breast cancer still caused 627,000 deaths in 2018. Since cancer is a multifactorial disease, it has become necessary to develop new molecular therapies that can target several relevant cellular processes at once. Ion channels are versatile regulators of several physiological- and pathophysiological-related mechanisms, including cancer-relevant processes such as tumor progression, apoptosis inhibition, proliferation, migration, invasion, and chemoresistance. Ion channels are the main regulators of cellular functions, conducting ions selectively through a pore-forming structure located in the plasma membrane, protein–protein interactions one of their main regulatory mechanisms. Among the different ion channel families, the Transient Receptor Potential (TRP) family stands out in the context of breast cancer since several members have been proposed as prognostic markers in this pathology. However, only a few approaches exist to block their specific activity during tumoral progress. In this article, we describe several TRP channels that have been involved in breast cancer progress with a particular focus on their binding partners that have also been described as drivers of breast cancer progression. Here, we propose disrupting these interactions as attractive and potential new therapeutic targets for treating this neoplastic disease.

## Introduction

### Breast Cancer Overview and Global Impact

Breast cancer is the second most frequent cancer type and the first cause of cancer-related deaths in women worldwide, with 627,000 deaths in 2018 (1). This neoplastic disease can be classified according to different parameters, providing further information about the tumor. In terms of diagnosis, breast tumors are histologically classified by their cellular origins in the mammary gland. If the tumor was originated by a ductal or lobular epithelial cell, it would be referred to as ductal or lobular carcinoma, respectively (2). For prognosis information, different molecular classifications have been developed. In this context, an important molecular classification is the "intrinsic subtype", which involves tumor classification according to the expression pattern of 50 genes related to cancer (PAM50). In PAM50, patients are sorted into six groups: Luminal A, Luminal B, HER2-enriched, Normal-like, Claudin-low, and Basal-like (3, 4). Another important molecular classification is the "surrogate intrinsic subtypes", which is the focus of this review. In this classification, tumors are grouped in five subtypes according to histologic features and expression of the Estrogen Receptor (ER), Progesterone Receptor (PR), and Human Epidermic Growth Factor Receptor 2 (HER2): Luminal A-like, Luminal B-like HER2−, Luminal B-like HER2+, HER2-enriched (non-luminal) and Triple-negative (TNBC). Luminal A-like, luminal B-like HER2- and luminal B-like HER2+ patients are the most common type, accounting for 75–80% of cases (4). Despite the above, these patients are usually treated with competitive inhibitors (also called hormonal therapy) that target the estrogen receptor, such as Tamoxifen or Fulvestrant. In cases where hormonal therapy fails, alternative treatments are employed, such as aromatase and mTOR inhibitors, which hinder and reduce estrogen synthesis. On the other hand, HER2-enriched tumors present increased levels of HER2, which is why the most efficient therapy consists of the use of Trastuzumab, a humanized monoclonal antibody against HER2. Even though Trastuzumab is the standard therapy for this group of patients, other HER2-related antibodies are available, such as Pertuzumab, an inhibitor of HER2 dimerization; Ado-trastuzumab (emtansine), an antibody–drug conjugate; and the tyrosine-kinase inhibitors lapatinib and neratinib, which inhibit both HER2 and the epithelial growth factor (EGFR) pathways (5). TNBC account for around 10–15% of breast cancer cases and are the hardest to treat (4). Since this group of patients is ER-, PR- and HER2-expression, there are no effective hormonal-based treatments or molecular therapies available. The treatment usually consists of surgery followed by chemotherapy and radiotherapy, whether individually or combined. However, its prognosis of TNBC remains poor (6).

### Calcium Signaling in Breast Cancer

Calcium ions (Ca^2+^) regulate several cellular processes in different organisms (7–9). Spatio-temporal changes in the cytoplasmic Ca^2+^ concentration occur in response to different stimuli that will be interpreted by the cell, promoting specific cellular outputs (10). Different molecular entities regulate Ca^2+^ homeostasis (11) and allow the cell to establish large Ca^2+^ concentration gradients (15,000–20,000 times) maintaining extremely low intracellular Ca^2+^ levels (close to 100 nM) compared to the extracellular medium (1–1.5 mM) (9, 12).

Ca^2+^ signaling modulates various cellular processes such as gene expression, cell cycle progression, cell migration, autophagy apoptosis (13), fertilization (14), and synapsis (15). Accordingly, aberrant intracellular Ca^2+^ signaling has been implicated in the development of different pathologies, such as neurodegenerative (16), metabolic (17, 18), and autoimmune disorders (19). In the context of neoplastic diseases, intracellular Ca^2+^-dependent signaling is involved in various processes that promote tumorigenesis and cancer progression, such as proliferation, migration, angiogenesis, and evasion of apoptosis (reviewed by 20–23).

Recent studies have highlighted specific regulators of Ca^2+^ signaling due to their role in the progression of breast cancer (24). These Ca^2+^ signaling regulators have been involved in controlling of proliferation, metastasis, cell death, and drug resistance. Therefore, several molecular entities that participate in these events have been suggested as possible therapeutic targets for breast cancer treatment (25). Among these regulators, the Transient Receptor Potential (TRP) channels superfamily has emerged as a novel and interesting target for therapeutic intervention in the context of breast cancer.

The TRP channels superfamily is characterized by sharing a common structure of six transmembrane segments and acting as polymodal cellular sensors for a broad spectrum of physical and chemical stimuli (26). TRP channels have been divided into seven families according to their sequence similarity (27, 28): TRPA (ankyrin), TRPC (canonical), TRPM (melastatin), TRPML (mucolipin), TRPN (no mechanoreceptor potential C or nompC), TRPP (polycystin or polycystic kidney disease), and TRPV (vanilloid and a proposed sister family, TRPVL) and recently a new member has been identified, TRPS (soromelastatin) (29).

Recent reports suggest the important role of TRP channels in different diseases, including cardiovascular, neurological, metabolic and neoplastic disorders (30). In cancer, changes in the expression and function of TRP channels are directly related to cellular processes that contribute to the progression of cancer, such as cell proliferation, differentiation, angiogenesis, migration, invasion and chemoresistance of cancer cells to apoptotic-induced cell death, promoting resistance to chemotherapy treatments (31). Several studies have shown the effect of modulating the activity of TRP channels in various cancer models using agonists and antagonists with or without the use of chemotherapeutic drugs, proposing them as excellent therapeutic targets (31–33). However, most of these agonists or antagonists are not specific and affect several TRP channels, and can impact their activity in other tissues, causing adverse effects (34).

**Figure 1:**
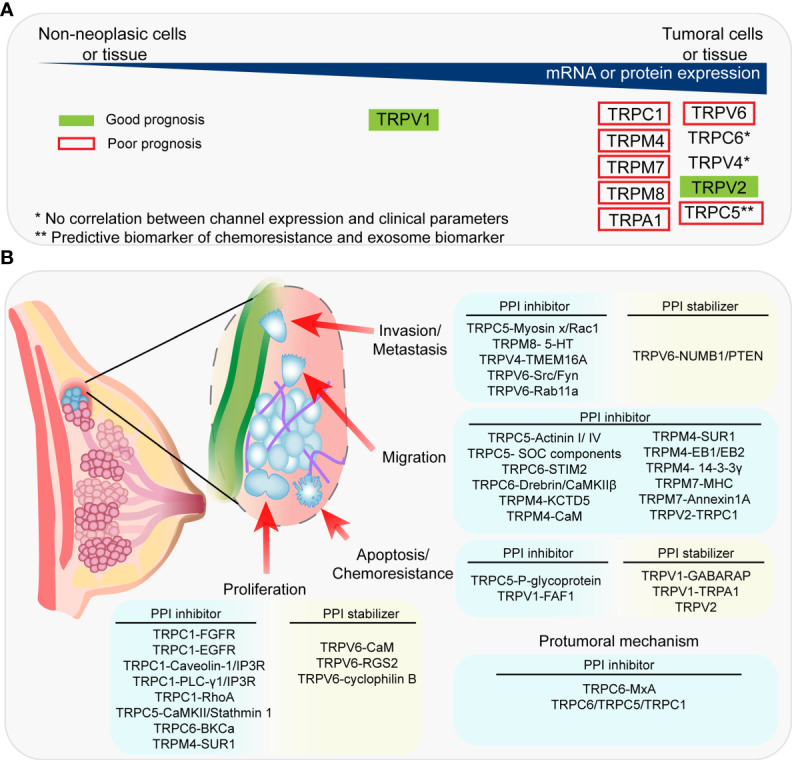
Association between the expression of TRP channels and clinical prognosis and PPIs proposed as therapeutic targets. **(A)** Summary of the expression of TRPC channels in tumoral *vs* non-tumoral tissue and the association between TRP channels and clinical prognosis. **(B)** Summary of the PPIs proposed as therapeutic targets and their associated-pro-tumoral processes.

In this context, TRP channels can form homomeric complexes or co-assemble into heteromeric functional tetramers with subunits of the same family or between different families (35). Also, these channels interact with an extensive network of different regulatory and structural proteins (TRIP Database: (36, 37). Drug discovery has been characterized by searching for molecular targets to identify new and potentially beneficial agents to selectively target disease-specific mechanisms and pathways involved in these diseases (38). Protein–protein interactions (PPIs) mediate and regulate most cellular functions and processes. Also, several diseases are caused by aberrant interactions between proteins and their regulators or effectors. Thus, the searching and design of drugs to disrupt PPIs have become increasingly more relevant (39). In the context of cancer, one of the approaches to study the impact of certain genes or proteins on the tumor phenotype and differentiate between causal and driver genes is to analyze their network of interactions and their effect on specific functions and pathways (40). Recent studies demonstrate that ion channels can be crucial in the organization of large macromolecular complexes serving for the regulation of diverse cellular processes (41, 42), as well as transduction nodes on which several signaling pathways might converge (43). Indeed, ion channels’ expression is altered in several types of cancer. Also, their activity has been considered of great importance in the development and progression of the disease, including breast cancer (44, 45). Interestingly, not only ion channels are dysregulated in cancer, but also their regulators, effectors, and other interacting proteins. Here we discuss the role of TRP channel interactors involved in breast cancer and propose the modulation of these interactions as therapeutic targets (**Figure 1** and **Supplementary Table 1**), opening a window for the design of more specific drugs against cancer.

## TRP Channels and Their Interactors in Breast Cancer

### TRPC1

TRPC1 is a non-selective cationic channel with a reported P_Ca_/P_Na_ ~1 in homotetrameric subunits (46–48). TRPC1 is expressed in diverse cell types such as smooth muscle, Central Nervous System (CNS) neurons, endothelial cells, platelets and salivary gland epithelial cells (46). Moreover, TRPC1 participates in endothelial permeability, vascular tone modulation (46), salivary fluid secretion (49), and synaptic plasticity (50). Mechanical stretch, receptor-dependent stimulation, diacylglycerol (DAG) (46, 51, 52) and ER-Ca^2+^ store depletion *via* STIM1 (53, 54) have been proposed as activation mechanisms.

The dysregulation in the activity of this channel has been reported in several type of cancer such as breast cancer, pancreatic cancer, lung cancer and others, where its overexpression has been related with poorly differentiated tumors and higher cell motility, proliferation and hypoxia-induced autophagy (55–58). Currently, it has been reported that TRPC1 promotes hypoxia-induced HIF1α-mediated EGFR signaling and expression of Epitelial–Mesenchymal Transition (EMT)-associated genes such as SNAIL in MDA-MB-468 cells (human TNBC cell line, Basal B). Consistently, TRPC1 expression is increased in Claudin-low human breast tumors and it has been associated with poor prognosis in patients with basal-like breast cancer (59). Importantly, TRPC1 is required for EGF-induced Ca^2+^ influx (48). Furthermore, TRPC1 interacts with FGFR1, and downregulation of TRPC1 inhibits proliferation in rat neural stem cells (60). Accordingly, TRPC1 silencing leads to decreased FGFR1 activation-induced Ca^2+^ signals. Both growth factor receptors, FGFR1 and EGFR, have crucial roles in cancer progression since their activation induces cell proliferation (61, 62). Moreover, FGFR1 expression is associated with resistance against endocrine therapy (63, 64), and EGFR is associated with poor prognosis in TNBC (61). These data suggest that TRPC1 might have important roles in FGFR1/EGFR-mediated breast cancer processes, which are still unaddressed. Since Ca^2+^ signaling is pivotal for these receptors’ downstream pathways, a novel therapeutic proposition might be to block TRPC1 activity or the functional interaction with either FGFR or EGFR.

TRPC1 interaction with Caveolin-1 is required for its proper plasma membrane targeting during Store-Operated Calcium Entry (SOCE) (65). Also, Caveolin-1 acts as a scaffold protein which mediates IP_3_R-TRPC1 interaction (66). Indeed, silencing Caveolin-1 decreases both SOCE and ER-Ca^2+^ depletion-induced association of TRPC1 to IP_3_Rs (67), indicating that Caveolin-1 plays an active role on Ca^2+^ homeostasis. The participation of Caveolin-1 in breast cancer has been extensively reviewed (68). Interestingly, Caveolin-1 participates in several breast cancer-progression events such as metastasis, cell cycle progression, and therapy resistance. Nevertheless, the current role of Caveolin-1 in breast cancer remains controversial due to different studies suggesting that this protein might act as a tumor suppressor. In contrast, others propose that it is instead an oncogene (68). Thus, the interplay between Caveolin-1 and TRPC1 in breast cancer is an open window for further research and future therapies. Similar to Caveolin-1, PLC-*γ*1 interacts with TRPC1 and IP_3_R during SOCE, suggesting a scaffolding role for this protein besides its IP3/DAG-production function (69).

PLC- *γ*1 overexpression has been proposed as a marker for metastasis development in early-stage luminal A and luminal B breast cancers (70). Interestingly, a synthetic peptide based on PLC-*γ*1 SH2 domains produces antitumoral effects in EGFR/HER-2 positive cells (71). Moreover, TRPC1 interacts with RhoA, a member of the Rho GTPases family. RhoA plays a crucial role in actin cytoskeleton dynamics, cell migration and contractility (72). Several authors have reported the opposite effects of RhoA in breast cancer models. Some studies state that RhoA prevents invasive processes (73, 74), while others propose that RhoA enhances cell invasiveness and proliferation of breast cancer cells (75, 76). This might be due to the fact that RhoA downstream effectors have opposing effects on pro-neoplastic processes (73). RhoA is suggested to promote TRPC1 association to IP_3_R during SOCE (77). Moreover, RhoA promotes the translocation of TRPC1 to the plasma membrane (77) and its activation (78), thus enhancing SOCE. Indeed, RhoA promotes cell migration of intestinal epithelial cells through the induction of SOCE *via* interaction with TRPC1 (78). Hence, TRPC1 might be an effector of RhoA activity, implying that this interaction’s specific disruption might impair TRPC1-dependent, breast cancer-associated pathways.

Interestingly, TRPC1 interaction with and activation by STIM1 confers a central role to this channel in the SOCE response contributing to the generation of the Store-Operated Currents (I_SOC_) (79). Moreover, TRPC1 interacts with several proteins that are implicated in the regulation of intracellular and ER Ca^2+^ concentrations, such as ORAI channels (80, 81), the SERCA pump (82, 83), PMCA (84), IP_3_R (82, 85), NCS-1 (86), and FKBP4 (87). Since Ca^2+^ signals are crucial for a myriad of cellular processes, it is not surprising that all these TRPC1-associated proteins have been proposed to play a role in breast cancer progression (88–93).

As the data suggests, the understanding of the dynamic cooperation between TRPC1-associated proteins mentioned above might shed light on the design and development of targeted therapeutics to selectively disrupt one or more of these interactions, depending on the differential features of the tumors.

### TRPC5

TRPC5 is a nonselective cation channel permeable to Ca^2+^ with a reported P_Ca_/P_Na_/P_Cs_ = 14.3/1.5/1, that can be activated following stimulation of receptors coupled to PLC in an intracellular Ca^2+^ dependent manner (94, 95). In cancer, this channel is mainly involved in angiogenesis and chemoresistance (96). Moreover, TRPC5 has been proposed as a predictive biomarker of chemoresistance in breast and colorectal cancer (97, 98). The expression of this ion channel is up-regulated together with P-glycoprotein, an efflux pump in Adriamycin-resistant breast cancer cells (MCF-7/ADM) (99, 100). In addition, the activation of this channel evokes Ca^2+^ currents, which induce P-glycoprotein over-expression, promoting chemoresistance (100). Moreover, TRPC5 was proposed as an exosome biomarker in breast cancer, which was demonstrated to have the capacity to transfer their chemoresistant phenotype to neighbor cells (101). Interestingly, this channel has been involved in Adriamycin resistance, mediating autophagy through the CaMKKβ/AMPKα/mTOR pathway (102).

TRPC5 interacts with several actin cytoskeleton regulators, such as Rac1 and several F-actin binding proteins such as Myosin X, α-actinin I, α -actinin IV and Neurabin II (103, 104). All these proteins have been involved in breast cancer progression. For instance, Myosin X is overexpressed in breast cancer tissue and breast cancer cell lines (MCF-7, BT-549 and MDA-MB-231) but not in non-cancerous breast cell lines (MCF-10A), promoting invadopodia formation, and metastasis in a Rac1-dependent manner (105). Rac1 is activated by TRPC5-mediated Ca^2+^ entry, promoting cell migration (103). Moreover, Rac1 is overexpressed and activated in the plasma membrane of tumor cells in aggressive breast cancer samples (106). These results suggest that TRPC5 interacts with Myosin X and Rac1, where the activation of Rac1 by TRPC5 or Myosin X might promote metastasis. Therefore, the disruption of these interactions could be beneficial for patients. On the other hand, α-actinin I and IV are actin binding proteins which localize in cell-cell adhesions, cell-matrix contact sites and in cellular protrusions, stabilizing these structures and linking membrane receptors with the cytoskeleton (107). The binding of α-actinin to Ca^2+^ reduces their affinity for actin (108). Moreover, the loss of α-actinin I in cell contact sites of TNBC cell lines (MDA-MB-231) promotes cell migration, whereas the same was reported for α-actinin IV in ER^+^/HER2^-^ cell lines (MCF-7 cells) (109, 110). These results suggest that α-actinin I and IV act as tumor suppressor proteins, while their Ca^2+^-dependent delocalization promotes migration in breast cancer cells. Although we cannot attribute these proteins’ delocalization only to TRPC5-dependent Ca^2+^ entry, promoting the localization of α-actinin I and IV to cell contact structures or disrupt their binding to Ca^2+^ could be useful in breast cancer treatment.

TRPC5 interacts with other proteins that modulate Ca^2+^ signaling, such as TRPC1, TRPC6, IP3R3 and STIM1. Moreover, all of these TRPC channels interact with the IP_3_R3 and STIM1 (111, 112), which could be an intrinsic characteristic of Store Operated Channels (SOC). Interestingly, the interaction between TRPC1, STIM1 and IP3R3 is relevant in Ca^2+^ signaling for cancer progression (55). Although the role of TRPC5 in cellular signaling through Ca^2+^ is still unclear, it is accepted that TRPC5-mediated Ca^2+^ entry activates CaMKIIβ inducing its phosphorylation in T287 (113), modification that has been described as increased in breast cancer tissue and metastasis (114). In addition, *in vitro* studies in MDA-MB-231 cells reported that CaMKIIβ promotes cell invasion (114). Therefore, the activation of CaMKIIβ through TRPC5-mediated Ca^2+^ entry might promote breast cancer progression, making of this interaction an interesting therapeutic target. In summary, although the mechanism by which TRPC5-mediated Ca^2+^ entry could promote cancer progression requires further investigation, the data suggests that disrupting interactions between TRPC5 and others SOC components (including IP_3_R3) involved in Ca^2+^ signaling could become a therapeutic strategy in breast cancer.

Stathmin1 is a protein which active or non-phosphorylated form mediates depolymerization of microtubules in late mitosis (115). Interestingly, it has been reported that tumors with high levels of this protein show enhanced proliferation, angiogenesis and immune response evasion, mainly in basal like subtypes of breast cancer (116). Moreover, increased expression of Stathmin1 correlates with poor prognosis for patients with breast cancer (117). Moreover, Stahmin1 is inhibited through CaMKII-mediated phosphorylation in a Ca^2+^-dependent manner, which in turn is activated by TRPC5 (113, 118). These results suggest that TRPC5, CaMKII and Stathmin1 could be part of a complex and a novel pathway to study as a therapeutic target.

### TRPC6

TRPC6 is a non-selective Ca^2+^ permeable channel with a P_Ca_/P_Na_ ~ 5, tightly receptor-operated and shows little basal activity (119). The main regulatory mechanism of TRPC6 is its activation by diacylglycerol (DAG) in response to the activation of G protein-coupled receptors. Additionally, other modulation mechanisms include regulation by Ca^2+^/Calmodulin (CaM) and binding of phosphoinositides (120). In humans, this channel is highly expressed in the lungs, placenta, ovary and spleen. It has important roles in regulating the heart and cardiopulmonary vasculature, podocyte functioning in the kidney, several neuronal processes, and several neoplastic diseases (121). In this context, TRPC6 was found upregulated in several types of cancer biopsies such as glioma, hepatocellular, gastric, esophageal and breast cancer, where it promotes migration invasion and proliferation (122–126). Specifically in breast cancer, studies in breast cancer cell lines showed high expression of TRPC6 in MCF-7 and MDA-MB-231 cells, whereas no expression was found in MCF-10A cells (122). Consistently, TRPC6 is the most overexpressed of all TRP channels in samples of human breast ductal adenocarcinomas compared to normal tissue. However, no correlation between TRPC6 expression and clinical parameters such as histological grade, tumor size, proliferation or invasiveness has been described (57). Although a precise role for TRPC6 in breast cancer has not been defined yet, there are studies relating to TRPC6 expression and activity with breast cancer progression in *in vitro* models. Moreover, TRPC6 promotes cell proliferation, migration and invasion in MCF-7 and MDA-MB-231 cell lines, an effect that is partially mediated by the modulation of surface expression of ORAI1 and ORAI3 Ca^2+^ channels (127). Recent studies showed that TRPC6 interacts and participates in conjunction with STIM2 to maintain cytosolic and endoplasmic reticulum Ca^2+^ concentrations in MCF-7 cells. Moreover, inhibition of TRPC6 activity leads to endoplasmic reticulum stress and caspase-3 activation (128). Conversely, TRPC6 associates with Large-Conductance Ca^2+^-Activated K^+^ (BKCa) channels, modulating their surface expression in podocytes (129). Interestingly, BKCa channels have been proposed as possible oncogenes in several types of cancer, including breast cancer, with augmented Ca^2+^ sensitivity, promoting shifts in the membrane potential that might promote tumor growth (130, 131). Thus, TRPC6-mediated BKCa modulation might contribute to tumor proliferation in breast cancer. TRPC6 also interacts with Fyn and Src tyrosine kinases, which positively regulate the activity of the channel through phosphorylation (132) and which have been reported as overexpressed in breast cancer cell lines and tissue (133, 134). Thus, Fyn and Src interaction with TRPC6 might be an important promoter of its activity and pro-tumoral effects in breast cancer. Moreover, TRPC6 activity is positively regulated by its interaction with the human myxovirus resistance protein 1 (MxA) (135), whose expression is higher in TNBC tumors than in other subtypes and is associated with a higher histologic grade (136). This data suggests that at least in TNBC, MxA-mediated TRPC6 increased activity might be a pro-tumoral mechanism promoting breast cancer progression. Another interesting interactor of TRPC6 is the actin-binding protein Drebrin, which, as we discuss previously, promotes the formation of cell protrusions in motile cells (104). Although a functional role for this interaction was not described, Drebrin interacts with and helps to localize and stabilize CaMKIIβ at actin filaments (137), which is a known positive regulator of TRPC6 activity (138), suggesting an interplay between Drebrin, CaMKIIβ and TRPC6, resulting in regulation of channel activity. Given the importance of both TRPC6 and Drebrin, this interaction and subsequent increase in TRPC6 activity might be one of the mechanisms by which Drebrin exerts its effects on breast cancer development, posing this interaction as an interesting and potential therapeutic target. Furthermore, TRPC6 interacts with several other TRPC family members of ion channels, such as TRPC1 and TRPC5 (139), downregulating the activity of the channel. As we mentioned above, these channels have also been reported as dysregulated in breast cancer. Thus, modulating the interplay between these TRPC channels might reduce Ca^2+^ influx and a subsequent reduction of tumor progression-associated processes.

### TRPM4

TRPM4 channel, is a non-selective cation calcium-activated channel permeable to monovalent ions, mainly Na^+^ and K^+^, with a permeability ratio P_Na_/P_Ca_ = 11, which is activated by intracellular Ca^2+^ (140–142). TRPM4 is broadly expressed in several tissues (141) and modulates several cell functions, including insulin secretion (143), immune response (144), cell migration and contractility (145, 146). TRPM4 also plays an important role in disease and several evidences has emerged linking the expression of TRPM4 with the increase of migration, invasion and proliferation of certain cancers, such as lymphoma (147), cervical (148), colorectal channel, prostate cancer (149–151) and others (152). Moreover, upregulation of this channel is associated with poor prognosis in large B cell lymphoma, breast cancer and endometrial carcinoma, while its overexpression increases the recurrence risk in prostate cancer (153–156). Recently, independent groups showed that TRPM4 is overexpressed in breast cancer samples of ER^+^/PR^+^ and triple-negative subtypes compared with normal breast tissue (153, 157). Moreover, TRPM4 expression is increased in IIB, IIIA, and IV stages and higher lymph node spread (N1–N2), according to the TNM classification (153, 157). In addition, TRPM4 gene was significantly associated with the expression of estrogen response and epithelial–mesenchymal transition (EMT) gene sets (157). As such, the identification of TRPM4 interactions might allow us to understand the biological role of TRPM4 in breast cancer and to broaden the search for molecular targets for its treatment. KCTD5 (Potassium Channel Tetramerization Domain-containing protein 5) interacts with TRPM4, regulating cell migration and contractility, by acting as a positive regulator of TRPM4 activity. Interestingly, KCTD5 mRNA is significantly upregulated in breast cancer tumors versus normal breast tissue samples (153), consistent with transcriptomics data in public databases (158). These data suggest that the increased expression of KCTD5 could be a key determinant of the malignancy and aggressiveness of these types of tumors, by interacting with TRPM4, enhancing the activity of the channel and thus, promoting TRPM4-dependent cell migration. Along the same lines, another interactor that acts as a positive regulator of channel activity is CaM which modulates the NFAT and Akt pathway promoting survival, proliferation and migration in several breast cancer cell lines in a Ca^2+^ dependent manner (159, 160). TRPM4 contains five CaM-binding sites and that by eliminating any three sites at the C-terminal, it strongly alters the activation current by decreasing Ca^2+^ sensitivity and modifying its voltage-dependent activation (161). Therefore, in the context of breast cancer, increased activity, and channel sensitivity due to TRPM4–CaM interaction could promote cell migration and contractility of tumor cells.

Another interesting interactor is sulfonylurea receptor-1 (SUR1), which forms a functional channel with TRPM4, presenting biophysical properties of TRPM4 and pharmacological properties of SUR1. Woo et al. showed that this TRPM4-SUR1 co-assembly has double affinity and sensitivity for CaM and Ca^2+^, respectively (162). SUR1 is a subunit of the inward-rectifier potassium ion channels Kir6.X (Kir6.1 and Kir6.2), whose association forms the K_ATP_ channels (163). SUR1 has been reported as overexpressed in lung cancer (164) and breast cancer (165). Studies using glibenclamide, a drug that binds SUR1, have shown a cytostatic effect on MDA-MB-231 cells, inhibiting cell cycle progression (166). Interestingly, recent studies suggest that this drug acts as an antagonist of the TRPM4–SUR1 interaction (167). Therefore, the interruption of this interaction might decrease cell migration and cell cycle progression, possibly through phosphorylation of CaMKII and decreased nuclear translocation of NFAT (168), which have also been implicated in cancer progression (169).

In the context of trafficking mechanisms and binding partners, two interesting TRPM4 interactors have been involved in the progression of breast cancer. First, EB1 and EB2 proteins that regulate the trafficking, surface expression and activity of TRPM4 (170). Interestingly, EB1 promotes the proliferation of cancer cells and have implicated EB1 in the tumorigenesis of breast cancer (171–173). For instance, EB1 expression correlates with higher histological grade, and the incidence of lymph node metastasis (171). Since the TRPM4–EB1 interaction has been shown to decrease FA turnover, and cell migration and invasion in murine melanoma cell lines (B16-F10 cells) (170), the disruption of the TRPM4–EB1 interaction might reduce the aggressiveness and metastatic capacity of breast cancer cells. Conversely, 14-3-3γ increases the surface expression of TRPM4 in HEK293T cells (174). The 14-3-3 protein family binds to the phosphorylated Ser/Thr motifs of its target proteins, regulating them through different mechanisms (175). Recently, the role of this family in age related-diseases such as neurodegenerative diseases and cancer, has been demonstrated (176). In breast cancer 14-3-3γ localizes in pseudopodia of MDA-MB-231 cells, where 14-3-3γ knockdown diminishes pseudopodia formation and cell migration (177). In summary, the TRPM4-14-3-3γ interaction could promote the expression of the channel on the surface, increasing its activity, which, given the data, could have a synergistic effect on cell migration of breast cancer cells. Therefore, interrupting the TRPM4-EB and TRPM4-14-3-3γ interactions could be favorable for breast cancer patients, reducing cancer cells’ migration and potential metastasis.

### TRPM7

TRPM7 is a member of the TRP family that permeates both Ca^2+^ and Mg^2+^. Moreover, free intracellular Mg^2+^ blocks this channel whereas ATP promotes its activation (178). In addition, TRPM7 possesses a unique C-terminal domain, which contains an active kinase domain (179). Interestingly, excision of the kinase domain enhances the TRPM7 ion channel activity (180). TRPM7 is broadly expressed in different tissues (181), and its ion channel function and kinase activity has been related to migration, cell proliferation and cell death in immune, hepatic, and different tumor cells (182). In the context of cancer, TRPM7 expression and activity has been associated with different types of cancer such as retinoblastoma (183), gastric cancer cells (184), head and neck carcinoma (185), and neuroblastoma (186, 187). In addition, TRPM7 expression is elevated in several histological subtypes, such as invasive ductal, invasive lobular (188) and in ER^-^ adenocarcinoma (189). Moreover, TRPM7 expression correlates with breast cancer progression and diminished survival of patients (190). Interestingly, the TRPM7 kinase domain directly phosphorylates the myosin heavy chain (MHC) (191, 192), an effect that was corroborated in MDA-MB-231 cells (189). Moreover, overexpression of TRPM7 increases migration in MCF-7 and MDA-MB-213 cells, while a truncated version that lacks the kinase domain has no effect on cell migration (189). TRPM7 silencing in MDA-MB-231 cells induces a redistribution of the actin cytoskeleton, promoting cell contraction (190). These data suggest that TRPM7 might regulate cell migration in breast cancer through phosphorylation of the MHC, facilitating invasiveness and metastasis. Thus, disrupting the interaction of TRPM7 with MHC, preventing its phosphorylation, may be a novel target to reduce metastasis formation in breast cancer.

Another TRPM7 phosphorylation target is the lipid-binding in a Ca^2+^-dependent manner protein Annexin 1A (193). Phosphorylation of Annexin 1A by TRPM7 stabilizes its interaction with membrane lipids (194). Interestingly, Annexin 1A is related to the pathogenesis of several cancer types (195), including breast cancer, where it has been proposed as an indicator of poor prognosis (196, 197). Annexin 1A promotes migration and metastasis in the highly invasive cell lines MDA-MB-231 and 4T1 (murine TNBC cell line) cells (196, 198). Interestingly, Annexin 1A depletion in MCF-7 cells increases the formation of focal adhesions and stress fibers, promoting a contractile phenotype, which is related to the effects observed when TRPM7 was absent. All together, these results suggest that TRPM7 might modulate cell migration and invasion of breast cancer through the regulation of Annexin 1A activity, suggesting this interaction as a possible therapeutic target against breast cancer.

Smad2 is a transcriptional regulator related to TGF-ß -induced EMT in several cancer types (199). Interestingly, Smad2 phosphorylation was reduced upon TRPM7 silencing (200). High levels of Smad2 phosphorylation are proposed as a marker of poor prognosis in breast cancer (201). Thus, TRPM7 inhibitors could decrease the levels of Smad2 phosphorylation, ameliorating the prognosis of patients.

In summary, the TRPM7 kinase activity is a novel pharmacological target to modulate invasiveness and metastasis of breast cancer, through the indirect regulation of the activity of its phosphorylation targets such as MHC, Annexin 1A or even Smad2.

### TRPM8

TRPM8 is a nonselective cationic ion channel with higher permeability for Ca^2+^ with a P_Ca_/P_Na_ ~1 (202). Notably, TRPM8 is a cold sensitive channel and is activated by cooling compounds such as menthol (203). Since TRPM8 is activated by cold temperatures, this channel is considered the molecular entity responsible for cold transduction in peripheral nerves (204). In the context of cancer, TRPM8 has been strongly involved in prostate cancer wherein it is related to tumor staging (205, 206). Moreover, this channel was found overexpressed in others types such as glioblastoma (207), pancreatic cancer (208), melanoma (209) and breast cancer (210). In breast cancer, TRPM8 expression is mainly upregulated in ER^+^ grade I adenocarcinomas (57, 211). Comparative analysis of TRPM8 mRNA levels shows higher expression in the highly invasive MDA-MB-231 cell line. Despite the precise analysis of TRPM8 expression in the different classifications of breast cancer, little is known about the participation of TRPM8 in the tumor pathology. For instance, TRPM8 silencing decreases migration and invasion in MDA-MB-231 cells, reducing the expression of EMT-related markers such as Akt, GSK-3ß phosphorylation and Snail expression. Conversely, TRPM8 overexpression in the low aggressive MCF-7 cell line increases invasion, migration and promotes EMT (212). The analysis of TRPM8 interacting proteins helps to hypothesize possible roles for this channel in the pathology of breast cancer. For instance, TRPM8 interacts with the Serotonin receptor 5-HT1B, enhancing the response of the channel to its agonists (213). In breast cancer, the 5-HT7 receptor is upregulated in triple negative cell lines such as the MDA-MB-231, HCC-1395, and Hs578T cells, increasing its invasion and proliferation through the PI3K/Akt pathway (214), an effect similar to that of TRPM8 (212), which suggests that 5-HT receptors might be a downstream effectors of TRPM8 activity.

### TRPV1

TRPV1 is a ligand-gated non-selective channel whose activation allows the influx of Ca^2 +^ and Na^+^ P_Ca_/P_Na_ = 9.6 (215, 216) and its selectivity depends on the nature and concentration of the agonist (217). TRPV1 can be activated by capsaicin and its analogues as well as by temperature, and pH changes (215) among others (216, 218–221). TRPV1 activity can be modulated by PKA (222), PKC (223) and CaMKII-dependent phosphorylation (224). Additionally, CaM and Inositol-4,5-biphosphate (PIP_2_) (225) binding have been reported to activate it. TRPV1 has been involved in processes such as thermoregulation and inflammatory nociception and is widely expressed in brain tissue, primary and secondary sensory neurons, arteriolar smooth muscle cells (226), skeletal muscle (227) and urothelium (228). TRPV1 has been associated with different diseases, including different types of cancer such as tongue squamous cell and prostate carcinoma (229, 230), hepatocellular carcinoma (231) and bladder carcinoma (232), skin cancer (233) and others (234–236). Although most studies report a tumor-suppressor role for TRPV1, its exact function in tumorigenesis is not clear. Increased expression of TRPV1 has been observed in all subtypes based on the molecular profile in breast cancer (237). In addition, TRPV1 expression pattern varies between cytoplasmic/membranous (165, 238) to a pattern in aggregates at the ER/Golgi, which was associated with a more aggressive tumoral stage (238).

Multiple studies have linked TRPV1 activity with an antitumoral role in breast cancer, promoting cell death (237, 239, 240) and decreased cell proliferation. Additionally, the activation of TRPV1 with agonists, modulators (MRS1477) and chemotherapeutic agents such as cisplatin induces cell death by apoptosis through depolarization of the mitochondrial membrane, ROS production and caspase activation (239, 240). Similarly, melatonin, in conjunction with the chemotherapeutic agent DOX, promotes apoptosis in MCF-7 cells through the activation of TRPV1 (241). Moreover, activation of TRPV1 using low doses of capsaicin may induce apoptosis in tumor cells, while higher doses of capsaicin activate necrosis (242). Wu et al. also determined that in MCF-7 cells, the expression levels of TRPV1 are decisive for capsaicin-induced cell death (243). Therefore, the use of TRPV1 agonists can be a complementary treatment to those currently used, promoting the sensitization of therapy-resistant tumors to cell death.

Fas-associated factor 1 (FAF1) is an adaptor protein for the Fas receptor and has been described to interact with TRPV1, reducing its response to capsaicin, heat and acid (244). A study performed in fibrosarcoma cells suggest that FAF1 acts as a negative regulator of cell death mediated by capsaicin-dependent activation of TRPV1. Moreover, FAF1 silencing increases fibrosarcoma cells susceptibility to apoptosis when cells were treated with capsaicin (245). However, in breast cancer, several studies have reported a tumor suppressor activity for FAF1, where this protein antagonizes Wnt signaling by stimulating β-catenin degradation (246). Additionally, FAF1 destabilizes TβRII on the cell surface limiting excessive TGF-β response, an effect that could supress metastasis formation (247). Furthermore, Xie et al. showed that there is a positive correlation between the survival of patients with metastasis-free breast cancer and the expression of FAF1. Therefore, the design of a drug to prevent the TRPV1/FAF1 interaction without affecting FAF1 tumor supressing activity could be a powerful tool in the treatment of breast cancer.

Another interesting interactor of TRPV1 is γ-aminobutyric acid type A (GABA_A_) receptor associated protein (GABARAP) where GABARAP increases TRPV1 expression, clustering at the cell surface and modulates TRPV1 gating and sensitivity (248). Moreover, GABARAP has been described as a tumor suppressor in breast cancer (249), where its mRNA and protein expression levels were significantly downregulated in invasive and ductal lobular carcinomas compared to normal breast tissue. Therefore, the design of a peptide that functionally emulates GABARAP that could promote its tumor suppressive role and increases the traffic of TRPV1 without altering its activity, could contribute to the treatment of breast cancer. Therefore, further studies into this interaction might represent an interesting new therapeutic approach.

TRPA1 is a nonselective cation channel permeable to Ca^2+^, Na^+^, and K^+^. TRPA1 detects a wide range of hazardous stimuli and is also involved in noxious cold and mechanical sensation (250). Different studies have demonstrated functional interaction between TRPA1 and TRPV1 (251, 252). Direct interaction of TRPV1 with TRPA1 exerts a Ca^2+^-dependent modulation on certain intrinsic properties of TRPA1 (253). Recently, it has been observed up-regulated expression of TRPA1 in breast cancer cells, promoting tolerance to oxidative stress in tumor cells. On the other hand, TRPA1 inhibition reduces tumor growth and improves sensitivity to chemotherapies (254). Interestingly, cell-permeable peptide that mimics the C-terminal of Tmem100-3Q, a modulator of the interaction between TRPA1 and TRPV1, selectively inhibit TRPA1-mediated activity in a TRPV1-dependent manner, generating a promising therapy for pain (255). Therefore, the design of peptides that promote the interaction of TRPV1–TRPA1 that might induce channel inhibition could be a potential treatment as has been observed.

### TRPV2

TRPV2 is a non-selective cation channel showing Ca^2+^ permeability, with a P_Ca_/P_Na_ ~3, and can be activated by high temperatures (>52 °C) (256), hypoosmolality (257), cell stretching (258), cannabinoids (259), among others (260, 261). TRPV2 is mainly expressed in the central and peripheral nervous system (256, 262–264) and at the subcellular level it is present in intracellular membranes (265, 266) and plasma membrane (267). TRPV2 act as a mechanosensor, osmosensor (257) and contributes to nociception and thermoregulation. However, studies performed in TRPV2 knock out mice suggest that it might participate in other activities (262), such as neurogenesis and gliomagenesis (268). TRPV2 has been involved in pathological processes, such as the development of cardiomyopathies (269) and cancer (270). In the context of cancer, the overexpression of channel is associated with well differentiated tumours and an increase of the survival in hepatocellular carcinoma, glioma and glioblastoma, while the opposite effect was observed in oesophageal carcinoma, urothelial carcinoma and gastric cancer (271–276). Increased TRPV2 expression has been observed in breast cancer samples, especially in TNBC subtype patients, where TRPV2 activation improved DOX uptake by tumor cells and was associated with better prognosis (277). Different studies have shown the formation of heteromers between TRPV1 and TRPV2 (278–280). Even though there are no studies directly linking their co-expression in breast cancer as indicated in the previous section, TRPV1, as well as TRPV2 have been described as markers of good prognosis. Therefore, enhancing their interaction and activation might be an interesting therapeutic approach.

Another interactor of TRPV2 is Acyl-CoA binding domain containing 3 (ACBD3), which mediates the TRPV2–PKA interaction and subsequent PKA-mediated phosphorylation (281). ACBD3 is involved in the maintenance of Golgi structure and function through its interaction with the integral membrane protein. However, a recent study shows that ACBD3 overexpression correlates with poor prognosis in breast cancer, where this protein promotes tumorigenesis by activating the Wnt/β-catenin signaling pathway (282). Although more studies are required on how the TRPV2–ACBD3 interaction might contribute to the modulation of channel activity or downstream signaling pathways in breast cancer contexts, it represents a novel and interesting therapeutic target to prevent disease progression.

### TRPV4

TRPV4 is a non-selective cationic channel with a reported relative permeability of P_Ca_/P_Na_ ~7 (283, 284). This channel is activated by multiple stimuli, such as osmotic changes, mechanical stretching, warm temperatures and arachidonic acid metabolites (285). It participates in several physiological processes such as vascular tone modulation, endothelial permeability, nociception and inflammatory responses in multiple organs (283, 285). Its expression has been reported in sensory and brain neurons, skeletal and smooth muscle as well as in epithelial tissue of several organs including the trachea, bladder, cornea, bile ducts and breast (283).

In the context of cancer, decreased TRPV4 expression has been reported in basal cell carcinoma, whereas an increase in its expression has been associated with poor survival in colorectal cancer (286, 287). Moreover, overexpression of this ion channel has been detected in breast cancer samples from human patients in comparison with normal tissue (288, 289). In this context, significantly higher expression was found in metastatic lesions compared with intraductal carcinomas (289) and in ER-negative compared with ER-positive samples (288). Moreover, TRPV4 expression has been associated with decreased survival of distant metastasis-free patients and enhanced EMT progression. This is consistent with its established role in non-metastatic murine breast cancer cell line (*i.e.* 4T07 cells), which involves the phosphorylation-dependent activation of Akt (289), pivotal kinase for EMT during breast carcinogenesis (290). Ca^2+^ signaling mediated by TRPV4 lead to phosphorylation-dependent Akt activation, causing a decrease of E-Cadherin expression (289). Interestingly, in keratinocytes it has been observed that TRPV4 forms a protein complex with β-catenin and E-cadherin in E-cadherin-dependent junctions, where TRPV4-mediated Ca^2+^ signals favours the maturation of these structures (291). This discrepancy might be explained by differences in the cellular model or due to alterations of regulatory mechanisms proper from neoplastic cells. Despite this, the presented data highly suggest that TRPV4 has an important role in breast cancer EMT progression *via* E-Cadherin regulation. Further studies are needed to unveil the possible functional association between these proteins in the context of breast cancer, which might grant valuable information for therapeutic approaches.

TRPV4 also interacts with Fyn and Src tyrosine kinase (292), protein associated with the progression and malignant features of diverse types of cancer (293). Similar to TRPV4, Fyn promotes EMT progression in breast cancer (294). Interestingly, Fyn-mediated phosphorylation of TRPV4 Tyr-253 residue is necessary for hypotonicity-induced Ca^2+^ entry, suggesting that Fyn is a positive regulator of TRPV4 (292). AQP5 associates with TRPV4 and is crucial for TRPV4-dependent Ca^2+^ influx promoted by hypotonic stimulus. AQP5 has been associated with breast cancer cells proliferation and migration (295) possibly by accelerating volume changes of the leading edge, contributing to actin cytoskeleton dynamics and cell shape changes during this process (296–298). Additionally, TRPV4 interaction with the Ca^2+^-activated Cl^-^ channel TMEM16A is also involved in volume regulation. Different studies show that TRPV4-mediated Ca^2+^ influx promotes TMEM16A activation, leading to Cl^−^ outward currents and subsequent water efflux (299, 300). It has been reported that TMEM16A is overexpressed in 78% of human breast cancer samples and that it modulates breast cancer progression through promotion of EGFR signaling in breast cancer cell lines (301). Thus, a possible interplay between Fyn, TRPV4, TMEM16A and AQP5 might occur during breast cancer progression and contribute to volume regulation during cell migration, leading to the development of a metastatic phenotype.

We previously mentioned the participation of IP_3_R3 and Caveolin-1 in breast cancer and their association with TRPC1. Interestingly, TRPV4 interaction with these proteins has been reported (302–304). Indeed, TRPV4 forms functional heterotetramers with TRPC1 (303) and is suggested to participate in SOCE (305). Moreover, the functional interaction between TRPC1 and TRPV4 participates in Ca^2+^ homeostasis of endothelial cells (305, 306). Thus, TRPV4 is crucial for the migration of breast cancer endothelial cells, an essential process for angiogenesis during tumor growth (307). In conclusion, the association of TRPV4 and TRPC1 could modulate tumor angiogenesis and involve Caveolin-1 and the IP3R3. Functional analysis of these interactions and their cellular outcomes remains to be explored to address this as a possible therapeutic target.

### TRPV6

TRPV6 is a Ca^2+^ channel with a P_Ca_/P_Na_ ~130 (308), regulated by intracellular Ca^2+^ in a Ca^2+^ and Ca^2+^/CaM-dependent manner (309). TRPV6 channels are widely expressed in absorptions epitheliums, exocrine glands, placenta, epididymal epithelium and in vestibular and cochlear tissues, where it mediates transcellular transport and maintains physiological levels of Ca^2+^ (310–313). In neoplastic diseases, TRPV6 channel has been reported as overexpressed in breast, colon, ovary, prostate and thyroid carcinomas promoting proliferation (314). Moreover, increased levels of this channel have been associated with high survival rates and better prognosis in cervical and oesophageal carcinomas, whereas the opposite effect has been reported in pancreatic cancer (315–317). In breast cancer, TRPV6 mRNA increased levels have been reported in tumor tissue and its expression has been correlated with poor prognosis for patients (57, 318). *In vitro* analysis has shown that TRPV6 silencing reduces breast cancer cell proliferation and promotes apoptosis (318). Interestingly, TRPV6 expression is promoted by the activation of vitamin D3, estrogen and androgen receptors. This induced overexpression increases the basal intracellular levels of Ca^2+^ in breast cancer cells, positively regulating gene expression via the CaM/Calcineurin/NFAT pathway, which modulates proliferation and apoptosis (314). Interestingly, this channel interacts with several proteins with important roles in breast cancer.

CaM, is a Ca^2+^ -sensing protein that regulates intracellular Ca^2+^ levels and has the capability to activate or inactivate several target proteins in a Ca^2+^-dependent manner (319). CaM induced the slow inactivation of TRPV6 channel by direct interaction (320) and modulates positively the NFAT and Akt pathways, promoting survival, proliferation and migration in several breast cancer cell lines in a Ca^2+^-dependent manner (159, 160). These results suggest that CaM inactivates TRPV6, reducing Ca^2+^ entry. Interestingly, in breast cancer, rises in Ca^2+^ levels might activate the NFAT and Akt pathways. Thus, uncoupling the TRPV6–CaM interaction could promote Ca^2+^ influx and activate those pathways. Thus, promoting this interaction could be beneficial for patients.

TRPV6-dependent Ca^2+^ currents are regulated by phosphorylation of the channel in tyrosine residues. For example, Fyn and Src are tyrosine kinases, members of the Src family, which regulate several processes, such as cell proliferation, survival, differentiation, motility and angiogenesis (321). Although Fyn interaction with TRPV6 was reported, the specific residues phosphorylated by Fyn remain unknown, whereas Src phosphorylates TRPV6 specifically in residues Y161 and Y162, promoting Ca^2+^ entry (322). In breast cancer, Fyn has been described as a predictive biomarker of tamoxifen response, participating in tamoxifen resistance (323). Moreover, this protein is involved in maintaining a mesenchymal phenotype in MDA-MB-231 cells (294). Interestingly, Src promotes cell growth and survival in MDA-MB-468, while in the MCF-7 cells, this protein promotes spreading and motility. Moreover, a gain of function of this protein promotes bone metastasis in mice models (324). Thus, these results suggest that tyrosine phosphorylation in TRPV6 promotes its activity, which could enhance breast cancer malignancy. For this reason, disruption of the interaction between these kinases and TRPV6 channels could be a therapeutic alternative in breast cancer.

TRPV6 also forms a complex with tumor suppressor proteins with relevance in cancer development such as Numb1 and phosphatidylinositol 3,4,5-trisphosphate 3-phosphatase (PTEN). Numb1 is a cell fate determinant protein, which stabilizes p53, preventing its ubiquitination. This protein interacts with TRPV6, decreasing cell Ca^2+^ influx, although its mechanism in unclear. Moreover, it has been described that in MCF-7 cells, Numb1 knockdown increases the expression of this channel, whereas TRPV6 knockdown increases Numb1 expression (325). On the other hand, PTEN is a protein phosphatase with tumor suppressor activity which mediates the PI (3–5) P_3_ to PI (4, 5) P_2_ conversion, promoting inhibition of the PI3K/Akt pathway (326). Interestingly, this phosphatase forms a complex with TRPV6 and Numb1 in the prostate cancer cell lines (DU145). Moreover, knockdown of PTEN causes a positive regulation between Numb1 and TRPV6 expression in DU145 cells (327). In breast cancer, the absence of Numb correlates with reduced disease-free survival in patients (328). A meta-analysis of generated data about PTEN suggests that the loss of this protein could predict more aggressive forms of breast cancer and worse prognosis for patients (329). Thus, these results suggest that loss of Numb and PTEN correlate with worse prognosis in breast cancer. For this reason, promoting the interaction between TRPV6, NUMB1 and PTEN could be useful as a therapeutic approach in breast cancer.

TRPV6 also interacts with accessory proteins with reported activity in breast cancer. For example, cyclophilin B is a peptidyl-prolyl cis-trans isomerase which promotes changes in protein conformation. This chaperone was identified as a TRPV6 accessory protein, promoting TRPV6-dependent Ca^2+^ influx (330). Moreover, the knockdown of cyclophilin B in ER^+^/HER2^−^ cell lines (T47D cells) downregulates the expression of several elements involved in cell proliferation, such as the progesterone and estrogen receptors (331). Another interesting protein is regulator of G-protein signaling 2 (RGS2) which corresponds to a negative regulator of G-protein coupled receptors (GPCR), increasing GTPase activity of Gα subunits and regulating several signaling pathways (332). This protein interacts with TRPV6 reducing its Na^+^ and Ca^2+^ currents, in a GPCR-independent manner (333). Interestingly, RGS2 is downregulated in MCF-7 cells but not in MCF-10A. Moreover, overexpression of this protein in MCF-7 cells reduces its proliferation (332). These results indicate that knockdown of cyclophilin B reduces TRPV6 currents and breast cancer cell proliferation. Moreover, overexpression of RGS2 reduces TRPV6 currents and breast cancer cell growth. Thus, we hypothesize that promoting these interactions could serve as a therapeutic approach in breast cancer.

An important component of TRPV6 trafficking machinery is Rab11. This protein regulates the budding, movement, and delivery of transport vesicles along different trafficking routes (334). Interestingly, Rab11 interacts with TRPV5 and TRPV6 mediating the trafficking of these proteins to the plasma membrane. Moreover, silencing of Rab11a promotes a decrease in membrane expression of both channels (309). In breast cancer cell lines, it has been described that Rab11a knockdown reduces proliferation, migration and invasion in an Akt-dependent manner (335, 336). These results reveal that downregulation of Rab11a reduces TRPV6-dependent Ca^2+^ currents and breast cancer cell malignancy. Thus, inhibiting the interaction between Rab11a and TRPV6 might constitute an interesting new target for breast cancer treatment.

### TRP Channels and STIM/ORAI Complex

As we described above, TRP channels exert multiple effects on breast cancer cells, at least in part, through the modulation of Ca^2+^ signaling. The SOCE response is crucial for Ca^2+^ homeostasis, and represents the main path for its influx in non-excitable cells. This response is triggered after endoplasmic reticulum Ca^2+^ depletion through the establishment of the STIM1/ORAI1 protein complex. The coupling between these proteins promotes the gating mechanism of the highly selective Ca^2+^ channel ORAI1, which leads to the establishment of Ca^2+^ Release-Activated Ca^2+^ Currents (I_CRAC_) (337). Although this current was the first associated to SOCE, other non-selective currents were identified as part of this homeostatic response as well, broadening the concept to I_SOC_ (338). This current has been associated with ORAI1 activity and with specific TRP channels, in particular with TRPC1 (80). Interestingly, SOCE has been widely associated with several types of cancer such as cervical, esophageal, breast, lung cancer and others (339). Moreover, a gain of function of this response has been related with a poor prognosis, bigger tumour size and poor differentiation (339). In addition, SOCE has been widely associated to breast cancer progression (340, 341). The expression of essential components of SOCE, such as ORAI1 and STIM1 are essential in mammary gland physiology during lactation, and also in breast cancer progression and invasion (342, 343). In addition to ORAI1, other ORAI isoforms have been linked to breast cancer. For instance, ORAI3 is a tumor promoting agent during cancer progression in ER+ malignant cells (344). Interestingly, increased levels of STIM1, TRPC1, ORAI1 and ORAI3 are correlated with poor prognosis of patients with aggressive basal breast cancer types, such as triple negative molecular subtypes (59, 345, 346). Although the exact mechanisms by which they could promote tumor progression are still not understood in detail, they might represent potential novel prognosis biomarkers in breast cancer.

In addition to TRPC1, other TRP channels have been related to SOCE and its signaling pathways. Several studies have described the physical and functional interaction between TRP channels and ORAI and STIM proteins (347). TRPC subfamily members are the most represented TRP channels forming complexes with SOCE components. In this context, TRPC3 (348) and TRPC6 (349) are proposed to interact with STIM1/ORAI1 complexes and promote Ca^2+^ entry by I_SOC_ and the subsequent activation of signaling pathways related to cancer progression (350). TRPC1/STIM1/ORAI1 complex activity might participate in metabolic regulation through Ca^2+^ dependent control of mitochondrial function and autophagy (351, 352). Other TRP subfamilies are also involved in Ca^2+^ entry through SOCE. TRPA1 interacts with the STIM1/ORAI1 complex, interfering with complex assembly, and decreasing the SOCE response (353). On the other hand, TRPV1, although it does not physically interact with ORAI1, exerts a functional modulation on this channel, particularly by promoting the Ca^2+^ dependent inactivation of ORAI1, leading to a decreased Ca^2+^ entry (354). Since these TRP channels and SOCE components have been previously proposed as prognosis biomarkers (56), its physical and functional interactions could be a potential target for the modulation of breast cancer progression. Other SOCE components are also related physically and functionally with TRP channels. STIM2 has been reported to interact with TRPC1 and TRPC6 in MCF-7 cells, modulating the resting Ca^2+^ content through SOCE activation (81, 128). TRPC1 and TRPC4 are activated by a longer isoform of STIM1, STIM1L. These interactions have different effect to that observed in the activation of SOCE related channels (355). While ORAI1 is preferentially activated by STIM1, TRPC1 is preferentially activated by STIM1L (356). Additionally, SOCE-Associated Regulatory Factor (SARAF), which is the main negative modulator of SOCE, interacts with either STIM1/ORAI1 complex and/or TRPC1 (357) decreasing in Ca^2+^ influx. Since several TRP channels interact with STIM1 and ORAI1, these could be considered a signaling hub in which TRP and ORAI channels’ activity might converge. Furthermore, since these physical and functional interactions between TRP channels and several SOCE components are reported to modulate Ca^2+^ entry and subsequent activation of pro-oncogenic processes in breast cancer cells, interrupting these interactions could be an exciting and novel strategy to attenuate cancer progression. TRPC channels and STIM/ORAI expression and interaction have been proposed as potential breast cancer prognosis biomarkers as proposed (56, 339). Further studies to explore the role of TRP channels and STIM1/ORAI1 complexes and how these are related to prognosis and their potential utility as biomarkers are necessary.

## Future Prospects for Other Possible Interactions of Ion Channels as Therapeutic Targets

As we previously discussed, the TRP channels interactome could be used as a novel therapeutic target in breast cancer by promoting or interrupting the discussed interactions, thus modulating channel activity and associated signaling pathways. As with others proteins, TRP channels can also be regulated by a plethora of post-translational modifications (PTM), which provide spatial–temporal control of several processes such as ion channel inactivation, activation, trafficking, endocytosis, and degradation (358–360). Interestingly, in several pathological conditions, including cancer, have been reported a dysregulation in the PTM balance. This dysregulation in PTM balance leads to several pathologies due to a gain or loss of function in crucial proteins (361–363). For example, tumor suppressors such as Rb, P53, and PTEN are targets of several PTM that mediated their inactivation or degradation, which promotes carcinogenesis (362). Although the relevance of PTM in cancer and TRP channels, further investigation is still required to identify new residues targets, the role of the modification in the channel, and the enzyme that mediates the PTM (359). Interestingly, only a few modifying enzymes have been reported as molecular partners of TRP channels such as Fyn, Src, PTEN, and KCTD5. However, all these presented relevant contributions in breast cancer and therefore could be exciting targets in molecular therapies (153, 294, 323, 324, 329). Further investigations are needed to identify more modifying enzymes and new putative targets in breast cancer and several others pathologies.

## Modulation of PPIS as a Therapeutic Strategy for Breast Cancer

**Figure 2:**
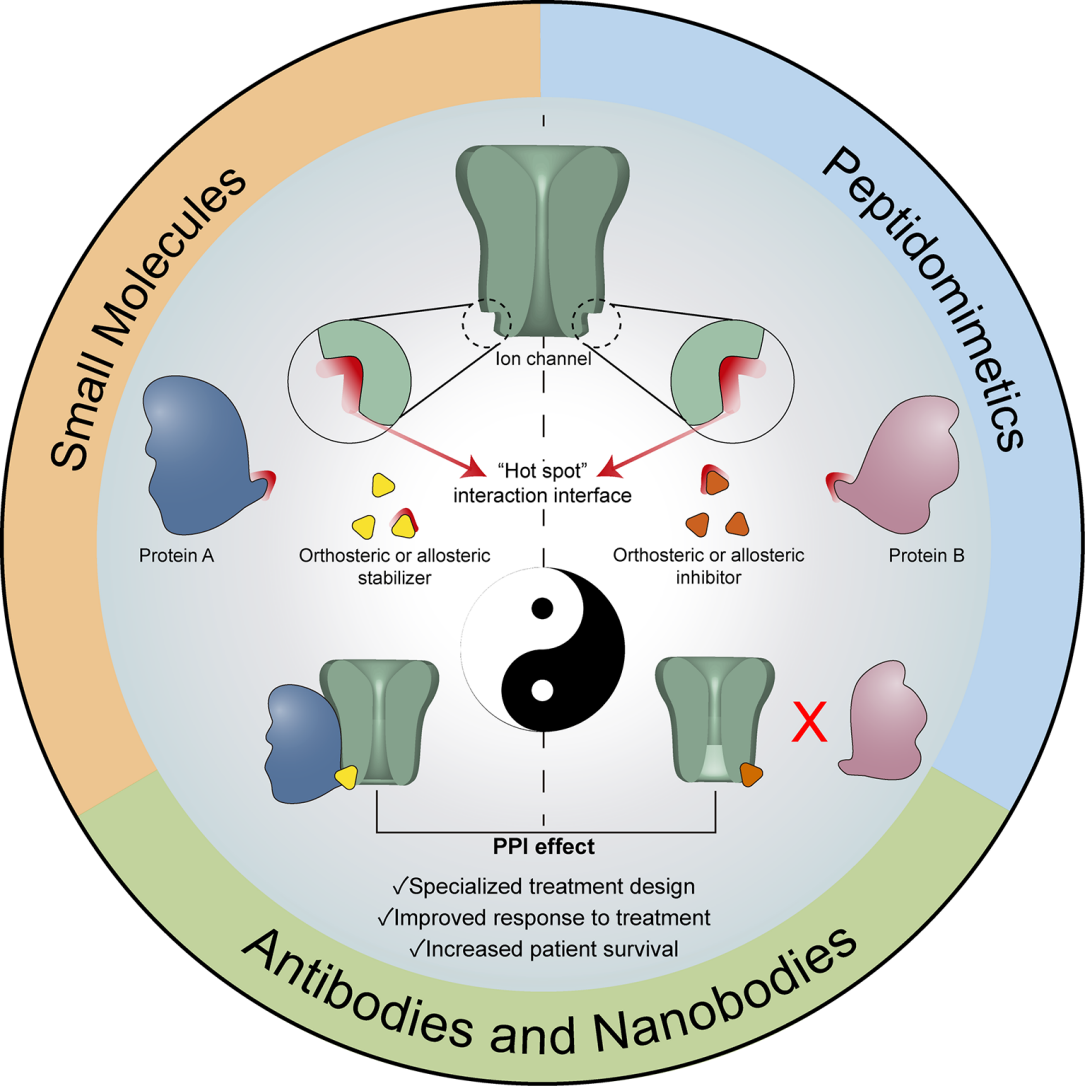
Modulation of ion channel-associated PPIs as therapeutic tool. Graphical representation of the modulation options for Ion channel-associated PPI, either through stabilizers or inhibitors of the interactions. All these strategies could constitute a useful complementary therapeutic tool contributing to increasing existing or personalized therapies.

Almost all breast cancer patients will receive at some point chemotherapy as either first-line treatment or as adjuvant/neoadjuvant, which will bring with it serious side effects for the patient (364). Given the systemic nature of the treatment and its low cell specificity and toxicity, chemotherapy can affect almost every organ in the body, including the heart, lungs, brain and kidneys (365, 366). These side effects, along with the fact that most times chemotherapy does not have a curative effect on breast cancer, but only prolongs the patient’s life and alleviates the disease-associated symptoms (365, 367) makes the discovery of new therapies that will only affect tumor cells an important field of research. As was described in the previous sections, there is a myriad of TRP channels that have been associated with several clinical parameters and diverse aspects of breast cancer progression, making them interesting therapeutic targets. Moreover, several interactors of these channels have also been described as important regulators in the development of breast cancer. The fact that the expression of both the TRP channels and their interactors are highly dysregulated in breast cancer suggests that the modulation of these interactions might provide new specific and selective targets for therapeutic interventions in the field of breast cancer research. Since peptides to modulate PPIs are highly specific in regards to their targets, affecting only the cellular pathways associated with a specific interaction and in cells where both the channel and the interactor are expressed, they are attractive candidates for *in vivo* treatment due to their minimal off-target effects (368). Nevertheless, compared to the targeting of enzymes or receptors, the therapeutic intervention of PPIs has been more challenging, partly due to the large and less structured interface of these interactions (369–372). Despite that, some of the current methods for PPIs modulation have been used as therapeutic strategy in breast cancer (**Figure 2**).

One of the main challenges that PPI modulators have faced is that most interactions encompassed relatively large contact areas. Nevertheless, subsequent research lead to the description of "hotspots," which are a small subset of amino acid residues contributing most of the binding free energy in PPIs (373). These hotspots are rich in Tyr, Trp, Leu, Ile, Phe, and Arg, where the amino acids Trp, Arg, and Tyr promote hydrogen bonds forming between the interacting proteins, contributing to π-interactions and binding free energy (373). This definition of hotspots has led to the design of small molecules or peptides that target this small number of amino acids to modulate PPIs. An early example of this was the design of Tirofiban, a mimetic of the Arg-Gly-Asp tripeptide epitope of fibrinogen that binds to the αIIbβ3 integrin approved by the FDA (374).

Another therapeutic challenge that these molecules might have is their poor cell/tissue specificity and membrane penetration capacity. In this context, the use of cell-penetrating peptides (CPPs) has provided an important tool to improve these aspects. CPPs are short peptides (less than 30 residues) with low cytotoxicity that have the ability to cross biological membranes, which can be coupled to bioactive molecules to transport them inside cells (375, 376). Interestingly, it has been demonstrated that several of these peptides have tumor homing capacities or might be modified to discriminate between tumoral and non-tumoral cells, thus, when coupled to PPIs-modulators, they would reduce undesired off-target effects and improve their cellular uptake (377–380). Several methods have been developed to provide CPPs with tumoral specificity, such as the use of peptides that naturally bind to tumor-specific cell surface molecules (380, 381), peptides that are inactive until modified by a cancer-associated protease or by properties of the tumoral microenvironment (382, 383) and those that take advantage of cellular mechanisms dysregulated in tumoral cells (384). As we can see, the use of CPPs is a major tool to provide PPI-modulators with tumoral specificity and low side effects.

Several small molecules have been designed and are being used for the targeting of pro-tumoral PPIs in breast cancer. Eribulin and Ixabepilone both interfere with the tubulin-α–tubulin-β interaction inhibiting microtubule growth, ultimately causing G2-M phase cell cycle arrest and cell death through apoptosis, have been used for years in the treatment of metastatic breast cancer patients (385–388). LCL161 and Birinapant are two among several SMAC mimetics being used in breast cancer treatment, which are modeled after the N-terminal AVPI tetrapeptide of Smac, which binds to the BIR domains of the inhibitor of apoptosis (IAP) proteins, thus interfering with this interaction (389, 390). These are just examples of PPIs and drugs used to target them in breast cancer treatment. Further discovery of small molecules is guaranteed by using current techniques, such as High Throughput/virtual Screening and Fragment-based methodologies (391). These approaches will be relevant to the discovery and development of new drugs targeting ion channel-associated PPIs. In this context, although modulators of TRPC channels-associated PPIs have not been approved for human treatment yet, several studies using *in vivo* models have shown the efficacy of using systemically-administered peptides to disrupt ion channels-associated PPIs to treat diseases with no observed side effects, and high specificity for their targets (392–395).

Despite their positive characteristics, small peptides do have several limitations, such as a lack of stability, relatively low affinity, poor cell-penetrability and short plasma half-life (396, 397). Given this, the use of antibodies has become an essential tool to interrupt PPIs and the fastest-growing and most successful class of therapeutic biologics, given their intrinsic ability to interact potently with proteins along a broader surface spectrum and various epitopes and their inherent stability (398). In this context, daclizumab is an FDA-approved antibody that blocks the interaction between IL-2 and its receptor, which has shown clinical success in patients with metastatic breast cancer when applied in combination with immune therapy (399). Atezolizumab is a monoclonal antibody against programmed death ligand-1 (PD-L1), which blocks its interaction with Programmed cell death protein 1 (PD-1) (400). In a recent study, the treatment with Atezolizumab plus nab-paclitaxel prolonged progression-free survival among patients with metastatic TNBC (401). Moreover, in a pilot study in women with early-stage breast cancer, the use of ipilimumab and FDA approved (for melanoma treatment) monoclonal antibody against CTLA-4, which blocks the interaction with its ligand CD86, showed potentially favorable intratumoral and systemic immunologic effects in combination with cryoablation (402). Although the use of antibodies to modulate PPIs has become a new and exciting tool to modulate PPIs, we acknowledge the relative difficulty of using them in the context of ion channels and their interactors due to the inability of antibodies to cross the plasma membrane, which is where most of the interactions in ion channels occur. The use and generation of recombinant antibodies might improve those difficulties. Additionally, the development of nanobodies, small single-domain antigen-binding antibody fragments, has helped to improve many of the problems that normal monoclonal antibodies have. The smaller size of nanobodies helps them to better penetrate deeper into the tumor and have a more precise targeting than normal IgGs (403–405). Moreover, nanobodies can be conjugated to CPPs to overcome their inability to penetrate the plasma membrane and thus be able to modulate pro-tumoral PPIs occurring inside the cell with great specificity without affecting the functionality of the involved proteins in other signaling pathways (404, 406, 407).

The more broadly used (and FDA approved) approaches to modulate PPIs are the small molecules and antibodies. However, other methods are being proved and improved to make them more suitable for clinical use, such as peptidomimetics, long peptides with a structure derived from existing peptides or protein domains. These molecules tend to mimic natural interaction and conserve a protein-like structure (391, 408). Although no peptidomimetics have been approved for breast cancer treatment, the major advances in the field of cell-penetrating peptides (CPPs), will encourage the development of therapeutic peptides and help to overcome some of the shortcomings that these molecules have as possible therapeutics (375, 409). Moreover, in the field of antibodies, nanobodies’ discovery and use should promote more stable and simpler molecules (410, 411). Also, aptamers can also be used to modulate PPIs (412, 413). Finally, the combination of several of these methods, such as the** **conjugation of peptides with small molecules, oligoribonucleotides, or antibodies as a possible alternative to overcome each type’s disadvantages, has gained interest in the last years, especially in oncology (396).

## Concluding Remarks

Cancer is a multifactorial disease, which progression depends on the dysregulation of several pathways involved in processes such as cell metabolism, immune evasion, avoiding cell death, inflammation, migration, and invasion. Interestingly, Ca^2+^ signaling is involved in many, if not all, of these processes. In this context, ion channels, especially TRP channel family members, are interesting proteins given their regulatory role on intracellular Ca^2+^ levels. Indeed, TRP channels expression and activity of several of them have been found commonly dysregulated in several types of cancer, including breast cancer. Hence, several TRP channels have been proposed as potential biomarkers of tumor progression and response to breast cancer treatment.

Interestingly, several proteins that interact with TRP channels have been reported as dysregulated in breast cancer, and thus, proposed as biomarkers for cancer progression. In this article, we have extensively reviewed the main TRP channels that might have a role in breast cancer progression, focusing on how their interaction with other reported biomarkers might constitute novel and exciting targets for intervention as possible therapies in this disease. Furthermore, we have presented several mechanisms by which these interactions might be regulating several aspects of breast cancer progression. However, although there are strong evidence and literature to support the interactions presented here, we are aware that most of them are based on reports in non-cancer models. Thus, further studies are needed to confirm these interactions and their possible pathological role in breast cancer. In conclusion, the identification and confirmation of new TRP-interactions, their role in breast cancer, and the development of drugs to modulate those interactions might provide new alternatives to help in the treatment of breast cancer patients, especially in those cases where there are no current treatments available or where treatments are non-selective and carry dangerous secondary effects with them.

## Data Availability Statement

The original contributions presented in the study are included in the article/**Supplementary Material**. Further inquiries can be directed to the corresponding author.

## Author Contributions

MS, DM, OO-S, IS, BL, PC, CT, MC, and OC wrote the article. MS designed and made the illustrations. All authors contributed to the article and approved the submitted version.

## Funding

FONDECYT Grant 1200917 and Millennium Nucleus of Ion Channel-Associated Diseases (MiNICAD) to OC fund this research. MiNICAD is a Millennium Nucleus supported by the Iniciativa Científica Milenio of the National Agency of Research and Development (ANID). FONDECYT Grant 1181263 funds to MC. CONICYT Doctoral Fellowship Program funds to BL (#21181611), PC (#21180306), IS (#21180245), and MS (#21191141) and DM (#21201941). OO-S is funded by the MiNICAD Postdoctoral Fellowship Program.

## Conflict of Interest

The authors declare that the research was conducted in the absence of any commercial or financial relationships that could be construed as a potential conflict of interest.
