# Supplementary material for: TRP Channels Interactome as a Novel Therapeutic Target in Breast Cancer
Source: Front Oncol. 2021 Jun 10;11:621614. doi: 10.3389/fonc.2021.621614 (PMC8222984; doi:10.3389/fonc.2021.621614)
Supplement: Supplementary file 1 [file Table_1.docx]

**Supplementary Table 1. Relevance of TRP channel interactors in breast cancer**

| TRP channel | Interactor | Relevance in breast cancer |
| --- | --- | --- |
| TRPC1 | Fibroblast growth factor receptor-1 protein (FGFR1)  (Fiorio Pla et al., 2005) | FGFR1 expression is associated with prognosis in ER+/HER2- primary breast cancer (Tomiguchi et al., 2016). Upregulation of EGFR signaling is correlated with tumor stroma remodelling and tumor recurrence in FGFR1-driven breast cancer (Holdman et al., 2015) |
|  | Caveolin1  (Pani et al., 2009) | Caveolin-1 inhibits breast cancer stem cells via c-Myc-mediated metabolic reprogramming (S. Wang et al., 2020) - Src-mediated phosphorylation, ubiquitination and degradation of Caveolin-1 promotes breast cancer cell stemness (Yoon, Kim, Kim, Jang, & Surh, 2019) |
|  | PLC-γ  (Tu, Chang, & Bikle, 2005) | PLC-γ-1 phosphorylation status is prognostic of metastatic risk in patients with early-stage Luminal-A and -B breast cancer subtypes (Lattanzio et al., 2019) |
|  | IP3R2 (Rosado & Sage, 2001)/  IP3R3 (Yuan et al., 2003) | Downregulation of IP3R decrease breast cancer cells migration (Mound et al., 2017) - IP3R3 silencing induces actin filaments reorganization in breast cancer cells by regulation of a ARHGAP18/RhoA/mDia1/FAK pathway (Vautrin-Glabik, Botia, Kischel, Ouadid-Ahidouch, & Rodat-Despoix, 2018) |
|  | STIM1  (J. J. López, Salido, Pariente, & Rosado, 2006) | STIM1 silencing reduces cell migration in TNBC (MDA-MB-132 and 4T1 cells) and metastasis in murine breast cancer model (Yang, Zhang, & Huang, 2009) |
|  | STIM2  (Berna-Erro, 2012) | STIM2 leads to breast cancer metastasis via NFAT1 / TGF-β1 to promote EMT.(Miao et al., 2019). |
|  | ORAI1  (Cheng, Liu, Ong, & Ambudkar, 2008) | ORAI1 silencing reduces cell migration in TNBC (MDA-MB-132 and 4T1 cells) and metastasis in murine breast cancer model (Yang et al., 2009) |
|  | ORAI3  (Berna-Erro, 2012) | Orai3 is overexpressed in breast cancer tissues, participating in their proliferation, cell cycle progression and survival (Faouzi, 2011; Hasna et al., 2018). |
|  | RhoA  (Mehta et al., 2003) | Some studies state that RhoA prevents invasive processes (Humphries et al., 2017; Kalpana, Figy, Yeung, & Yeung, 2019) while others propose that RhoA enhances cell invasiveness and proliferation of breast cancer cells (Daubriac et al., 2018; Pillé et al., 2005) |
|  | PMCA  (Singh, Liu, Tang, Zhu, & Ambudkar, 2002) | PMCA2 regulates breast cancer cell proliferation and sensitivity to doxorubicin (Peters et al., 2016) and promotes HER2-mediated breast cancer (Jeong et al., 2016) |
|  | NCS-1  (Hannan, Kabbani, Paspalas, & Levenson, 2008) | NCS-1 expression is higher in basal breast cancers and regulates calcium influx and cytotoxic responses to doxorubicin (Bong, Robitaille, Milevskiy, Roberts-Thomson, & Monteith, 2020). Promotes aggressive behaviour and predicts survival of patients (Moore, England, Ehrlich, & Rimm, 2017) |
|  | SERCA2/ SERCA3 (Redondo, Jardin, Lopez, Salido, & Rosado, 2008) | Inhibition of SERCA by thapsigargin analogs induces cell death via ER Ca^2+^ depletion and the unfolded protein response (Sehgal et al., 2017) |
|  | FKBP4  (E. López, Berna-Erro, Salido, Rosado, & Redondo, 2013; Sinkins, Goel, Estacion, & Schilling, 2004) | FKBP4 is a malignant indicator in luminal A subtype of breast cancer (Xiong et al., 2020) - FKBP4 connects mTORC2 and PI3K to activate the PDK1/Akt-dependent cell proliferation signaling in breast cancer (Mangé et al., 2019) |
| TRPC5 | Rac1  (Tian et al., 2010) | Rac1 is overexpressed and activated in the plasma membrane of tumor cells in aggressive breast cancer samples (Schnelzer et al., 2000) |
|  | Myosin X  (Goel, Sinkins, Keightley, Kinter, & Schilling, 2005) | Myosin X was found overexpressed in breast cancer tissue (Cao et al., 2014) |
|  | α-actinin I  (Goel et al., 2005) | The loss of α-actinin I in cell contact sites of MDA-MB-231 cells promotes cell migration (Kovac, Mäkelä, & Vallenius, 2018) |
|  | α -actinin IV  (Goel et al., 2005) | The loss of α-actinin IV in cell contact sites of MCF-7 cells promotes cell migration (Hsu & Kao, 2013) |
|  | Drebrin1  (Goel et al., 2005) | DBN1 is an independent prognostic marker in luminal breast cancer due to its association with the response to endocrine therapy (Alfarsi et al., 2020). |
|  | CaMKIIβ  (Puram et al., 2011) | Phosphorylation in T287 that has been described as increased in breast cancer tissue and metastasis. In vitro studies in MDA-MB-231 cells report that CaMKIIβ promotes cell invasion (Chi et al., 2016) |
|  | TARBP2  (Zimmermann et al., 2014) | Overexpression of TARBP2 correlates with downregulation of tumor suppressors APP and ZNF395 and enhanced metastasis (Goodarzi et al., 2014). TARBP2 induces resistance to Tamoxifen in a SOX2-dependent manner in MCF-7 cells and breast tumors (M. Y. Wang et al., 2019). |
|  | NCS1  (Hui et al., 2006) | Overexpression of NCS1 promotes migration and metastasis in MDA-MB-231 (Apasu et al., 2019). NCS1 is overexpressed in basal like breast cancer, where it acts as a poor prognosis and doxorubicin resistance biomarker (Bong et al., 2020). NCS1 has also been found positively correlated with complete response to Taxane-based chemotherapy through its direct interactions (Moore et al., 2018) |
|  | TRPC1  (Strübing, Krapivinsky, Krapivinsky, & Clapham, 2001) | Overexpression promotes proliferation in breast cancer tissue and MCF-7 (Elzamzamy, Penner, & Hazlehurst, 2020) |
|  | TRPC6  (Shi, Ju, Saleh, Albert, & Large, 2010) | Silencing of this protein reduces proliferation, migration and invasion in MDA-MB-231 and MCF-7 cells (Jardin et al., 2018) |
|  | IP3R3  (Tang et al., 2001) | Its interaction with BKCa promotes proliferation in MCF-7 cells (Mound, Rodat-Despoix, Bougarn, Ouadid-Ahidouch, & Matifat, 2013) |
|  | STIM1  (Yuan, Zeng, Huang, Worley, & Muallem, 2007) | Overexpression promotes metastasis in triple negative breast cancer cells (MDA-MB-132 and 4T1 cells) (Yang et al., 2009) |
|  | Stathmin1  (Greka, Navarro, Oancea, Duggan, & Clapham, 2003) | High levels of this protein show enhanced proliferation, angiogenesis and immune response evasion, mainly in basal like subtypes of breast cancer (Askeland et al., 2020). Stathmin1 correlates with poor prognosis for breast cancer patients (Askeland et al., 2020) |
| TRPC6 | BKCa (E. Y. Kim, Alvarez-Baron, & Dryer, 2009) | Metastatic breast cancer cells exhibit increased BKCa channel activity, leading to greater invasiveness and transendothelial migration, both of which could be attenuated by blocking BKCa.(Khaitan et al., 2009) |
|  | Fyn (Hisatsune et al., 2004) | FYN promotes breast cancer progression through  epithelial-mesenchymal transition (Y. G. Xie et al., 2016) |
|  | Src (Hisatsune et al., 2004) | Screening a spectrum of breast cancer cell lines with dasatinib highlighted preferential sensitivity to Src inhibition in basal-like breast cancers. Src also plays a role in resistance of HER2+ breast cancer to the HER2-targeted antibody trastuzumab (Mayer & Krop, 2010) |
|  | MxA (Lussier et al., 2005) | MxA expression was higher in TNBC tumors than in other subtypes. High MxA levels are associated with a higher histologic grade and abundant tumor infiltrating lymphocytes (Y. A. Kim et al., 2016) |
|  | Drebrin1 (Goel et al., 2005) | DBN1 is an independent prognostic marker in luminal breast cancer due to its association with the response to endocrine therapy (Alfarsi et al., 2020). |
| TRPM4 | KCTD5  (Rivas et al., 2020a) | KCTD5 mRNA is significantly upregulated in breast cancer tumors compared to normal tissue samples (Rivas et al., 2020b) |
|  | Calmodulin (CaM)  (Nilius et al., 2005) | Modulates the NFAT and AKT pathway promoting survival, proliferation and migration in several breast cancer cell lines in a Ca^2+^-dependent manner (Coticchia, Revankar, Deb, Dickson, & Johnson, 2009; Deb, Coticchia, & Dickson, 2004) |
|  | SUR1  (Woo, Kwon, Ivanov, Gerzanich, & Simard, 2013) | SUR1 is overexpressed in breast cancer (Uhlen et al., 2017). |
|  | EB1  (Blanco et al., 2019) | EB1 expression correlates with higher histological grade and metastasis (Dong et al., 2010). EB1 promotes the proliferation and tumorigenesis of breast cancer (Dong et al., 2010; Fujii et al., 2005; Y. Wang et al., 2005). |
|  | 14-3-3gamma  (Cho et al., 2014) | 14-3-3γ localizes in pseudopodia of MDA-MB-231 cells and its knockdown diminishes the pseudopodia formation and cell migration (Hiraoka et al., 2019) |
|  | TRPC3  (Park et al., 2008) | In MDA-MB-231 cells, Ca^2+^ influx through TRPC3 via RAS4/Ras-MAPK pathway promotes proliferation and apoptosis resistance (Y. Wang, Qi, Qi, & Tsang, 2019) |
|  | ENaC  (E. C. Kim, Choi, Lim, Yeon, & Lee, 2013) | γENaC induces a chronic inflammatory response with a potential protumoral effect in breast cancer cell lines (Amara, Ivy, Myles, & Tiriveedhi, 2016) |
| TRPM7 | Myosin Heavy Chain IIA (MHCIIa)  (Clark et al., 2006; Middelbeek et al., 2016) | MHCIIa promotes invasion and migration of breast cancer cell lines (Guilbert et al., 2013) |
|  | Annexin 1a  (Dorovkov & Ryazanov, 2004; Zhao et al., 2015) | Annexin 1A expression is upregulated in basal-like breast cancer cell lines, regulating metastasis and TGF-ß signaling (de Graauw et al., 2010) |
|  | Smad2  (L. Fang et al., 2014) | Smad2 phosphorylation is increased in patients, suggesting a poor prognosis of survival (de Kruijf et al., 2013) |
| TRPM8 | 5-HT 1β  (Vinuela-Fernandez et al., 2014) | 5-HT receptor expression is up-regulated in the highly invasive cell line MDA-MB-231. 5-HT receptor is implicated in cell invasion and tumor growth (Gautam et al., 2016) |
| TRPV1 | Cbl  (S. Li et al., 2011) | Cbl is a predictor of favourable prognosis in breast cancer (Daniels et al., 2019; W. Li et al., 2018; Liu et al., 2020; L. Xu et al., 2017) |
|  | EGFR  (Bode et al., 2009) | EGFR is over-expressed in metastatic breast cancer and TNBC (Masuda, 2012). |
|  | FAF1  (S. Kim et al., 2006) | A positive correlation has been observed between the survival of patients with metastasis-free breast cancer and FAF1 expression (F. Xie et al., 2017) |
|  | GABARAP  (Laínez et al., 2010) | GABARAP has been described as a tumor suppressor in breast cancer where their mRNA and protein expression levels were significantly downregulated in invasive and ductal lobular carcinomas compared to normal breast tissue (Klebig et al., 2005) |
|  | TRPA1  (Akopian, Ruparel, Jeske, & Hargreaves, 2007; Salas, Hargreaves, & Akopian, 2009) | TRPA1 is overexpressed in breast cancer tissue promoting tolerance to oxidative stress of tumor cells and its inhibition reduces tumor growth and improves sensitivity to chemotherapy (Takahashi et al., 2018) |
| TRPV2 | TRPV1  (Hellwig, Albrecht, Harteneck, Schultz, & Schaefer, 2005; Liapi & Wood, 2005; Rutter, Ma, Leveridge, & Bonnert, 2005) | TRPV1 activity exerts an antitumoral role in breast cancer, promoting cell death (Nazıroğlu et al., 2017; Nur, Nazıroğlu, & Deveci, 2017; Weber et al., 2016) and decreased cell proliferation. |
|  | ACBD3  (Stokes, Shimoda, Koblan-Huberson, Adra, & Turner, 2004) | ACBD3 overexpression correlates with poor prognosis in breast cancer, promoting tumorigenesis via activation of Wnt/β-catenin signalling (Huang et al., 2018) |
| TRPV4 | β-catenin/E-Cadherin  (Kida et al., 2012; Sokabe, Fukumi-Tominaga, Yonemura, Mizuno, & Tominaga, 2010) | Protein complex that plays crucial roles in EMT and metastatic behaviour (Corso et al., 2020) |
|  | Fyn  (H. Xu et al., 2003) | Fyn promotes breast cancer progression through EMT (Y. G. Xie et al., 2016) and promotes a mesenchymal phenotype in breast cancer cells (Lee et al., 2018) |
|  | AQP5  (Liu et al., 2006) | AQP5 expression is a marker for proliferation and migration of breast cancer cells (Jung, 2011), and a prognostic marker in triple-negative breast cancer (Zhu et al., 2018) |
|  | Caveolin1  (Ma et al., 2010) | Caveolin-1 inhibits breast cancer stem cells via c-Myc-mediated metabolic reprogramming (S. Wang et al., 2020) - Src-mediated phosphorylation, ubiquitination and degradation of Caveolin-1 promotes breast cancer cell stemness (Yoon et al., 2019) |
|  | TMEM16A  (Takayama, Shibasaki, Suzuki, Yamanaka, & Tominaga, 2014) | TMEM16A induces EGFR-dependent signaling pathway activation and breast cancer cells proliferation and migration (Britschgi et al., 2013; H. Wang et al., 2017) |
|  | IP3R3  (Fernandes et al., 2008; Garcia-Elias, Lorenzo, Vicente, & Valverde, 2008) | Downregulation of IP3R decrease breast cancer cells migration (Mound et al., 2017) - IP3R3 silencing induces actin filaments reorganization in breast cancer cells by regulation of a ARHGAP18/RhoA/mDia1/FAK pathway (Vautrin-Glabik et al., 2018) |
|  | Calmodulin  (Niemeyer, Bergs, Wissenbach, Flockerzi, & Trost, 2001) | Calmodulin modulates the NFAT and AKT pathways promoting survival, proliferation and migration in several breast cancer cell lines in a Ca^2+^-dependent manner (Coticchia et al., 2009; Deb et al., 2004) |
| TRPV6 | Fyn  (Sternfeld et al., 2007) | Fyn has been described as a predictive biomarker of tamoxifen response, participating in tamoxifen resistance (Elias et al., 2015).This protein is involved in maintaining the mesenchymal phenotype of MDA-MB-231 cells (Lee et al., 2018) |
|  | Src  (Sternfeld et al., 2007) | Src promotes cell growth and survival in triple negative and EGFR positive breast cancer cells (MDA-MB-468), while in MCF-7 cell this protein promotes spreading and motility. Moreover, a gain of function of this protein promotes bone metastasis in mice models (Finn, 2008) |
|  | Numb1  (S. Y. Kim et al., 2013) | The absence of Numb1 correlates with reduced disease-free survival in patients with a basal like phenotype (Rennstam et al., 2010) |
|  | PTEN  (S. Y. Kim et al., 2014) | PTEN loss of function correlates with a more aggressive behaviour and poor prognosis for patients (S. Li et al., 2017) |
|  | Cyclophilin B  (Stumpf et al., 2008) | Knockdown of cyclophin B in T47D cells downregulates the expression of several elements involved in cell proliferation, such as the progesterone and estrogen receptors (F. Fang, Flegler, Du, Lin, & Clevenger, 2009) |
|  | RGS2  (Schoeber et al., 2006) | RGS2 is downregulated in MCF-7 cells but not in MCF-10A. RGS2 overexpression in MCF-7 cells reduces cell growth (Lyu et al., 2015) |
|  | Rab11a  (van de Graaf, Hoenderop, & Bindels, 2006) | Rab11a knockdown reduces proliferation, migration, and invasion in an AKT dependent manner (W. Li, Li, Fan, & Liu, 2017) |

**References**

Akopian, A. N., Ruparel, N. B., Jeske, N. A., & Hargreaves, K. M. (2007). Transient receptor potential TRPA1 channel desensitization in sensory neurons is agonist dependent and regulated by TRPV1-directed internalization. *J Physiol, 583*(Pt 1), 175-193. doi:10.1113/jphysiol.2007.133231

Alfarsi, L. H., El Ansari, R., Masisi, B. K., Parks, R., Mohammed, O. J., Ellis, I. O., . . . Green, A. R. (2020). Integrated Analysis of Key Differentially Expressed Genes Identifies DBN1 as a Predictive Marker of Response to Endocrine Therapy in Luminal Breast Cancer. *Cancers (Basel), 12*(6). doi:10.3390/cancers12061549

Amara, S., Ivy, M. T., Myles, E. L., & Tiriveedhi, V. (2016). Sodium channel γENaC mediates IL-17 synergized high salt induced inflammatory stress in breast cancer cells. *Cell Immunol, 302*, 1-10. doi:10.1016/j.cellimm.2015.12.007

Apasu, J. E., Schuette, D., LaRanger, R., Steinle, J. A., Nguyen, L. D., Grosshans, H. K., . . . Ehrlich, B. E. (2019). Neuronal calcium sensor 1 (NCS1) promotes motility and metastatic spread of breast cancer cells in vitro and in vivo. *FASEB J, 33*(4), 4802-4813. doi:10.1096/fj.201802004R

Askeland, C., Wik, E., Finne, K., Birkeland, E., Arnes, J. B., Collett, K., . . . Akslen, L. A. (2020). Stathmin expression associates with vascular and immune responses in aggressive breast cancer subgroups. *Sci Rep, 10*(1), 2914. doi:10.1038/s41598-020-59728-3

Berna-Erro, A. a. G. C. a. D. N. a. G. L. J. a. S. G. M. a. R. J. A. (2012). Capacitative and non-capacitative signaling complexes in human platelets. *Biochimica et Biophysica Acta - Molecular Cell Research, 1823*(8), 1242--1251. doi:10.1016/j.bbamcr.2012.05.023

Blanco, C., Morales, D., Mogollones, I., Vergara-Jaque, A., Vargas, C., Álvarez, A., . . . Cerda, O. (2019). EB1- and EB2-dependent anterograde trafficking of TRPM4 regulates focal adhesion turnover and cell invasion. *FASEB J, 33*(8), 9434-9452. doi:10.1096/fj.201900136R

Bode, A. M., Cho, Y. Y., Zheng, D., Zhu, F., Ericson, M. E., Ma, W. Y., . . . Dong, Z. (2009). Transient receptor potential type vanilloid 1 suppresses skin carcinogenesis. *Cancer Res, 69*(3), 905-913. doi:10.1158/0008-5472.CAN-08-3263

Bong, A. H. L., Robitaille, M., Milevskiy, M. J. G., Roberts-Thomson, S. J., & Monteith, G. R. (2020). NCS-1 expression is higher in basal breast cancers and regulates calcium influx and cytotoxic responses to doxorubicin. *Mol Oncol, 14*(1), 87-104. doi:10.1002/1878-0261.12589

Britschgi, A., Bill, A., Brinkhaus, H., Rothwell, C., Clay, I., Duss, S., . . . Bentires-Alj, M. (2013). Calcium-activated chloride channel ANO1 promotes breast cancer progression by activating EGFR and CAMK signaling. *Proc Natl Acad Sci U S A, 110*(11), E1026-1034. doi:10.1073/pnas.1217072110

Cao, R., Chen, J., Zhang, X., Zhai, Y., Qing, X., Xing, W., . . . Zhu, X. (2014). Elevated expression of myosin X in tumours contributes to breast cancer aggressiveness and metastasis. *Br J Cancer, 111*(3), 539-550. doi:10.1038/bjc.2014.298

Cheng, K. T., Liu, X., Ong, H. L., & Ambudkar, I. S. (2008). Functional requirement for Orai1 in store-operated TRPC1-STIM1 channels. *J Biol Chem, 283*(19), 12935-12940. doi:10.1074/jbc.C800008200

Chi, M., Evans, H., Gilchrist, J., Mayhew, J., Hoffman, A., Pearsall, E. A., . . . Skelding, K. A. (2016). Phosphorylation of calcium/calmodulin-stimulated protein kinase II at T286 enhances invasion and migration of human breast cancer cells. *Sci Rep, 6*, 33132. doi:10.1038/srep33132

Cho, C. H., Kim, E., Lee, Y. S., Yarishkin, O., Yoo, J. C., Park, J. Y., . . . Hwang, E. M. (2014). Depletion of 14-3-3γ reduces the surface expression of Transient Receptor Potential Melastatin 4b (TRPM4b) channels and attenuates TRPM4b-mediated glutamate-induced neuronal cell death. *Mol Brain, 7*, 52. doi:10.1186/s13041-014-0052-3

Clark, K., Langeslag, M., van Leeuwen, B., Ran, L., Ryazanov, A. G., Figdor, C. G., . . . van Leeuwen, F. N. (2006). TRPM7, a novel regulator of actomyosin contractility and cell adhesion. *EMBO J, 25*(2), 290-301. doi:10.1038/sj.emboj.7600931

Corso, G., Figueiredo, J., De Angelis, S. P., Corso, F., Girardi, A., Pereira, J., . . . Gandini, S. (2020). E-cadherin deregulation in breast cancer. *J Cell Mol Med, 24*(11), 5930-5936. doi:10.1111/jcmm.15140

Coticchia, C. M., Revankar, C. M., Deb, T. B., Dickson, R. B., & Johnson, M. D. (2009). Calmodulin modulates Akt activity in human breast cancer cell lines. *Breast Cancer Res Treat, 115*(3), 545-560. doi:10.1007/s10549-008-0097-z

Daniels, S. R., Liyasova, M., Kales, S. C., Nau, M. M., Ryan, P. E., Green, J. E., & Lipkowitz, S. (2019). Loss of function Cbl-c mutations in solid tumors. *PLoS One, 14*(7), e0219143. doi:10.1371/journal.pone.0219143

Daubriac, J., Han, S., Grahovac, J., Smith, E., Hosein, A., Buchanan, M., . . . Boucher, Y. (2018). The crosstalk between breast carcinoma-associated fibroblasts and cancer cells promotes RhoA-dependent invasion via IGF-1 and PAI-1. *Oncotarget, 9*(12), 10375-10387. doi:10.18632/oncotarget.23735

de Graauw, M., van Miltenburg, M. H., Schmidt, M. K., Pont, C., Lalai, R., Kartopawiro, J., . . . van de Water, B. (2010). Annexin A1 regulates TGF-beta signaling and promotes metastasis formation of basal-like breast cancer cells. *Proc Natl Acad Sci U S A, 107*(14), 6340-6345. doi:10.1073/pnas.0913360107

de Kruijf, E. M., Dekker, T. J. A., Hawinkels, L., Putter, H., Smit, V., Kroep, J. R., . . . Mesker, W. E. (2013). The prognostic role of TGF-β signaling pathway in breast cancer patients. *Ann Oncol, 24*(2), 384-390. doi:10.1093/annonc/mds333

Deb, T. B., Coticchia, C. M., & Dickson, R. B. (2004). Calmodulin-mediated activation of Akt regulates survival of c-Myc-overexpressing mouse mammary carcinoma cells. *J Biol Chem, 279*(37), 38903-38911. doi:10.1074/jbc.M405314200

Dong, X., Liu, F., Sun, L., Liu, M., Li, D., Su, D., . . . Zhou, J. (2010). Oncogenic function of microtubule end-binding protein 1 in breast cancer. *J Pathol, 220*(3), 361-369. doi:10.1002/path.2662

Dorovkov, M. V., & Ryazanov, A. G. (2004). Phosphorylation of annexin I by TRPM7 channel-kinase. *J Biol Chem, 279*(49), 50643-50646. doi:10.1074/jbc.C400441200

Elias, D., Vever, H., Lænkholm, A. V., Gjerstorff, M. F., Yde, C. W., Lykkesfeldt, A. E., & Ditzel, H. J. (2015). Gene expression profiling identifies FYN as an important molecule in tamoxifen resistance and a predictor of early recurrence in patients treated with endocrine therapy. *Oncogene, 34*(15), 1919-1927. doi:10.1038/onc.2014.138

Elzamzamy, O. M., Penner, R., & Hazlehurst, L. A. (2020). The Role of TRPC1 in Modulating Cancer Progression. *Cells, 9*(2). doi:10.3390/cells9020388

Fang, F., Flegler, A. J., Du, P., Lin, S., & Clevenger, C. V. (2009). Expression of cyclophilin B is associated with malignant progression and regulation of genes implicated in the pathogenesis of breast cancer. *Am J Pathol, 174*(1), 297-308. doi:10.2353/ajpath.2009.080753

Fang, L., Huang, C., Meng, X., Wu, B., Ma, T., Liu, X., . . . Li, J. (2014). TGF-β1-elevated TRPM7 channel regulates collagen expression in hepatic stellate cells via TGF-β1/Smad pathway. *Toxicol Appl Pharmacol, 280*(2), 335-344. doi:10.1016/j.taap.2014.08.006

Faouzi, M. a. H. F. a. P. M. a. A. A. a. S. H. a. O.-A. H. (2011). Down-regulation of Orai3 arrests cell-cycle progression and induces apoptosis in breast cancer cells but not in normal breast epithelial cells. *Journal of Cellular Physiology, 226*(2), 542--551. doi:10.1002/jcp.22363

Fernandes, J., Lorenzo, I. M., Andrade, Y. N., Garcia-Elias, A., Serra, S. A., Fernández-Fernández, J. M., & Valverde, M. A. (2008). IP3 sensitizes TRPV4 channel to the mechano- and osmotransducing messenger 5'-6'-epoxyeicosatrienoic acid. *J Cell Biol, 181*(1), 143-155. doi:10.1083/jcb.200712058

Finn, R. S. (2008). Targeting Src in breast cancer. *Ann Oncol, 19*(8), 1379-1386. doi:10.1093/annonc/mdn291

Fiorio Pla, A., Maric, D., Brazer, S. C., Giacobini, P., Liu, X., Chang, Y. H., . . . Barker, J. L. (2005). Canonical transient receptor potential 1 plays a role in basic fibroblast growth factor (bFGF)/FGF receptor-1-induced Ca2+ entry and embryonic rat neural stem cell proliferation. *J Neurosci, 25*(10), 2687-2701. doi:10.1523/JNEUROSCI.0951-04.2005

Fujii, K., Kondo, T., Yokoo, H., Yamada, T., Iwatsuki, K., & Hirohashi, S. (2005). Proteomic study of human hepatocellular carcinoma using two-dimensional difference gel electrophoresis with saturation cysteine dye. *Proteomics, 5*(5), 1411-1422. doi:10.1002/pmic.200401004

Garcia-Elias, A., Lorenzo, I. M., Vicente, R., & Valverde, M. A. (2008). IP3 receptor binds to and sensitizes TRPV4 channel to osmotic stimuli via a calmodulin-binding site. *J Biol Chem, 283*(46), 31284-31288. doi:10.1074/jbc.C800184200

Gautam, J., Banskota, S., Regmi, S. C., Ahn, S., Jeon, Y. H., Jeong, H., . . . Kim, J. A. (2016). Tryptophan hydroxylase 1 and 5-HT(7) receptor preferentially expressed in triple-negative breast cancer promote cancer progression through autocrine serotonin signaling. *Mol Cancer, 15*(1), 75. doi:10.1186/s12943-016-0559-6

Goel, M., Sinkins, W., Keightley, A., Kinter, M., & Schilling, W. P. (2005). Proteomic analysis of TRPC5- and TRPC6-binding partners reveals interaction with the plasmalemmal Na(+)/K(+)-ATPase. *Pflugers Arch, 451*(1), 87-98. doi:10.1007/s00424-005-1454-y

Goodarzi, H., Zhang, S., Buss, C. G., Fish, L., Tavazoie, S., & Tavazoie, S. F. (2014). Metastasis-suppressor transcript destabilization through TARBP2 binding of mRNA hairpins. *Nature, 513*(7517), 256-260. doi:10.1038/nature13466

Greka, A., Navarro, B., Oancea, E., Duggan, A., & Clapham, D. E. (2003). TRPC5 is a regulator of hippocampal neurite length and growth cone morphology. *Nat Neurosci, 6*(8), 837-845. doi:10.1038/nn1092

Guilbert, A., Gautier, M., Dhennin-Duthille, I., Rybarczyk, P., Sahni, J., Sevestre, H., . . . Ouadid-Ahidouch, H. (2013). Transient receptor potential melastatin 7 is involved in oestrogen receptor-negative metastatic breast cancer cells migration through its kinase domain. *Eur J Cancer, 49*(17), 3694-3707. doi:10.1016/j.ejca.2013.07.008

Hannan, M. A., Kabbani, N., Paspalas, C. D., & Levenson, R. (2008). Interaction with dopamine D2 receptor enhances expression of transient receptor potential channel 1 at the cell surface. *Biochim Biophys Acta, 1778*(4), 974-982. doi:10.1016/j.bbamem.2008.01.011

Hasna, J., Hague, F., Rodat-Despoix, L., Geerts, D., Leroy, C., Tulasne, D., . . . Kischel, P. (2018). Orai3 calcium channel and resistance to chemotherapy in breast cancer cells: the p53 connection. *Cell Death Differ, 25*(4), 693-707. doi:10.1038/s41418-017-0007-1

Hellwig, N., Albrecht, N., Harteneck, C., Schultz, G., & Schaefer, M. (2005). Homo- and heteromeric assembly of TRPV channel subunits. *J Cell Sci, 118*(Pt 5), 917-928. doi:10.1242/jcs.01675

Hiraoka, E., Mimae, T., Ito, M., Kadoya, T., Miyata, Y., Ito, A., & Okada, M. (2019). Breast cancer cell motility is promoted by 14-3-3γ. *Breast Cancer, 26*(5), 581-593. doi:10.1007/s12282-019-00957-4

Hisatsune, C., Kuroda, Y., Nakamura, K., Inoue, T., Nakamura, T., Michikawa, T., . . . Mikoshiba, K. (2004). Regulation of TRPC6 channel activity by tyrosine phosphorylation. *J Biol Chem, 279*(18), 18887-18894. doi:10.1074/jbc.M311274200

Holdman, X. B., Welte, T., Rajapakshe, K., Pond, A., Coarfa, C., Mo, Q., . . . Rosen, J. M. (2015). Upregulation of EGFR signaling is correlated with tumor stroma remodeling and tumor recurrence in FGFR1-driven breast cancer. *Breast Cancer Res, 17*, 141. doi:10.1186/s13058-015-0649-1

Hsu, K. S., & Kao, H. Y. (2013). Alpha-actinin 4 and tumorigenesis of breast cancer. *Vitam Horm, 93*, 323-351. doi:10.1016/B978-0-12-416673-8.00005-8

Huang, Y., Yang, L., Pei, Y. Y., Wang, J., Wu, H., Yuan, J., & Wang, L. (2018). Overexpressed ACBD3 has prognostic value in human breast cancer and promotes the self-renewal potential of breast cancer cells by activating the Wnt/beta-catenin signaling pathway. *Exp Cell Res, 363*(1), 39-47. doi:10.1016/j.yexcr.2018.01.003

Hui, H., McHugh, D., Hannan, M., Zeng, F., Xu, S. Z., Khan, S. U., . . . Weiss, J. L. (2006). Calcium-sensing mechanism in TRPC5 channels contributing to retardation of neurite outgrowth. *J Physiol, 572*(Pt 1), 165-172. doi:10.1113/jphysiol.2005.102889

Humphries, B., Wang, Z., Li, Y., Jhan, J. R., Jiang, Y., & Yang, C. (2017). ARHGAP18 Downregulation by miR-200b Suppresses Metastasis of Triple-Negative Breast Cancer by Enhancing Activation of RhoA. *Cancer Res, 77*(15), 4051-4064. doi:10.1158/0008-5472.Can-16-3141

Jardin, I., Diez-Bello, R., Lopez, J. J., Redondo, P. C., Salido, G. M., Smani, T., & Rosado, J. A. (2018). TRPC6 Channels Are Required for Proliferation, Migration and Invasion of Breast Cancer Cell Lines by Modulation of Orai1 and Orai3 Surface Exposure. *Cancers (Basel), 10*(9). doi:10.3390/cancers10090331

Jeong, J., VanHouten, J. N., Dann, P., Kim, W., Sullivan, C., Yu, H., . . . Wysolmerski, J. J. (2016). PMCA2 regulates HER2 protein kinase localization and signaling and promotes HER2-mediated breast cancer. *Proc Natl Acad Sci U S A, 113*(3), E282-290. doi:10.1073/pnas.1516138113

Jung, H. J. a. P. J. Y. a. J. H. S. a. K. T. H. (2011). Aquaporin-5: A marker protein for proliferation and migration of human breast cancer cells. *PLoS ONE, 6*(12). doi:10.1371/journal.pone.0028492

Kalpana, G., Figy, C., Yeung, M., & Yeung, K. C. (2019). Reduced RhoA expression enhances breast cancer metastasis with a concomitant increase in CCR5 and CXCR4 chemokines signaling. *Sci Rep, 9*(1), 16351. doi:10.1038/s41598-019-52746-w

Khaitan, D., Sankpal, U. T., Weksler, B., Meister, E. A., Romero, I. A., Couraud, P. O., & Ningaraj, N. S. (2009). Role of KCNMA1 gene in breast cancer invasion and metastasis to brain. *BMC Cancer, 9*, 258. doi:10.1186/1471-2407-9-258

Kida, N., Sokabe, T., Kashio, M., Haruna, K., Mizuno, Y., Suga, Y., . . . Tominaga, M. (2012). Importance of transient receptor potential vanilloid 4 (TRPV4) in epidermal barrier function in human skin keratinocytes. *Pflugers Arch, 463*(5), 715-725. doi:10.1007/s00424-012-1081-3

Kim, E. C., Choi, S. K., Lim, M., Yeon, S. I., & Lee, Y. H. (2013). Role of endogenous ENaC and TRP channels in the myogenic response of rat posterior cerebral arteries. *PLoS One, 8*(12), e84194. doi:10.1371/journal.pone.0084194

Kim, E. Y., Alvarez-Baron, C. P., & Dryer, S. E. (2009). Canonical transient receptor potential channel (TRPC)3 and TRPC6 associate with large-conductance Ca2+-activated K+ (BKCa) channels: role in BKCa trafficking to the surface of cultured podocytes. *Mol Pharmacol, 75*(3), 466-477. doi:10.1124/mol.108.051912

Kim, S., Kang, C., Shin, C. Y., Hwang, S. W., Yang, Y. D., Shim, W. S., . . . Oh, U. (2006). TRPV1 recapitulates native capsaicin receptor in sensory neurons in association with Fas-associated factor 1. *J Neurosci, 26*(9), 2403-2412. doi:10.1523/JNEUROSCI.4691-05.2006

Kim, S. Y., Hong, C., Wie, J., Kim, E., Kim, B. J., Ha, K., . . . So, I. (2014). Reciprocal positive regulation between TRPV6 and NUMB in PTEN-deficient prostate cancer cells. *Biochem Biophys Res Commun, 447*(1), 192-196. doi:10.1016/j.bbrc.2014.03.123

Kim, S. Y., Yang, D., Myeong, J., Ha, K., Kim, S. H., Park, E. J., . . . So, I. (2013). Regulation of calcium influx and signaling pathway in cancer cells via TRPV6-Numb1 interaction. *Cell Calcium, 53*(2), 102-111. doi:10.1016/j.ceca.2012.10.005

Kim, Y. A., Lee, H. J., Heo, S. H., Park, H. S., Park, S. Y., Bang, W., . . . Gong, G. (2016). MxA expression is associated with tumor-infiltrating lymphocytes and is a prognostic factor in triple-negative breast cancer. *Breast Cancer Res Treat, 156*(3), 597-606. doi:10.1007/s10549-016-3786-z

Klebig, C., Seitz, S., Arnold, W., Deutschmann, N., Pacyna-Gengelbach, M., Scherneck, S., & Petersen, I. (2005). Characterization of {gamma}-aminobutyric acid type A receptor-associated protein, a novel tumor suppressor, showing reduced expression in breast cancer. *Cancer Res, 65*(2), 394-400.

Kovac, B., Mäkelä, T. P., & Vallenius, T. (2018). Increased α-actinin-1 destabilizes E-cadherin-based adhesions and associates with poor prognosis in basal-like breast cancer. *PLoS One, 13*(5), e0196986. doi:10.1371/journal.pone.0196986

Lattanzio, R., Iezzi, M., Sala, G., Tinari, N., Falasca, M., Alberti, S., . . . Piantelli, M. (2019). PLC-gamma-1 phosphorylation status is prognostic of metastatic risk in patients with early-stage Luminal-A and -B breast cancer subtypes. *BMC Cancer, 19*(1), 747. doi:10.1186/s12885-019-5949-x

Laínez, S., Valente, P., Ontoria-Oviedo, I., Estévez-Herrera, J., Camprubí-Robles, M., Ferrer-Montiel, A., & Planells-Cases, R. (2010). GABAA receptor associated protein (GABARAP) modulates TRPV1 expression and channel function and desensitization. *FASEB J, 24*(6), 1958-1970. doi:10.1096/fj.09-151472

Lee, G. H., Yoo, K. C., An, Y., Lee, H. J., Lee, M., Uddin, N., . . . Lee, S. J. (2018). FYN promotes mesenchymal phenotypes of basal type breast cancer cells through STAT5/NOTCH2 signaling node. *Oncogene, 37*(14), 1857-1868. doi:10.1038/s41388-017-0114-y

Li, S., Bode, A. M., Zhu, F., Liu, K., Zhang, J., Kim, M. O., . . . Dong, Z. (2011). TRPV1-antagonist AMG9810 promotes mouse skin tumorigenesis through EGFR/Akt signaling. *Carcinogenesis, 32*(5), 779-785. doi:10.1093/carcin/bgr037

Li, S., Shen, Y., Wang, M., Yang, J., Lv, M., Li, P., & Chen, Z. (2017). Loss of PTEN expression in breast cancer: association with clinicopathological characteristics and prognosis. *Oncotarget, 8*(19), 32043-32054. doi:10.18632/oncotarget.16761

Li, W., Li, G., Fan, Z., & Liu, T. (2017). Tumor-suppressive microRNA-452 inhibits migration and invasion of breast cancer cells by directly targeting RAB11A. *Oncol Lett, 14*(2), 2559-2565. doi:10.3892/ol.2017.6426

Li, W., Xu, L., Che, X., Li, H., Zhang, Y., Song, N., . . . Teng, Y. (2018). C-Cbl reverses HER2-mediated tamoxifen resistance in human breast cancer cells. *BMC Cancer, 18*(1), 507. doi:10.1186/s12885-018-4387-5

Liapi, A., & Wood, J. N. (2005). Extensive co-localization and heteromultimer formation of the vanilloid receptor-like protein TRPV2 and the capsaicin receptor TRPV1 in the adult rat cerebral cortex. *Eur J Neurosci, 22*(4), 825-834. doi:10.1111/j.1460-9568.2005.04270.x

Liu, X., Bandyopadhyay, B. C., Bandyopadhyay, B., Nakamoto, T., Singh, B., Liedtke, W., . . . Ambudkar, I. (2006). A role for AQP5 in activation of TRPV4 by hypotonicity: concerted involvement of AQP5 and TRPV4 in regulation of cell volume recovery. *J Biol Chem, 281*(22), 15485-15495. doi:10.1074/jbc.M600549200

Liu, X., Teng, Y., Wu, X., Li, Z., Bao, B., Liu, Y., . . . Zhang, L. (2020). The E3 Ubiquitin Ligase Cbl-b Predicts Favorable Prognosis in Breast Cancer. *Front Oncol, 10*, 695. doi:10.3389/fonc.2020.00695

Lussier, M. P., Cayouette, S., Lepage, P. K., Bernier, C. L., Francoeur, N., St-Hilaire, M., . . . Boulay, G. (2005). MxA, a member of the dynamin superfamily, interacts with the ankyrin-like repeat domain of TRPC. *J Biol Chem, 280*(19), 19393-19400. doi:10.1074/jbc.M500391200

Lyu, J. H., Park, D. W., Huang, B., Kang, S. H., Lee, S. J., Lee, C., . . . Baek, S. H. (2015). RGS2 suppresses breast cancer cell growth via a MCPIP1-dependent pathway. *J Cell Biochem, 116*(2), 260-267. doi:10.1002/jcb.24964

López, E., Berna-Erro, A., Salido, G. M., Rosado, J. A., & Redondo, P. C. (2013). FKBP52 is involved in the regulation of SOCE channels in the human platelets and MEG 01 cells. *Biochim Biophys Acta, 1833*(3), 652-662. doi:10.1016/j.bbamcr.2012.11.029

López, J. J., Salido, G. M., Pariente, J. A., & Rosado, J. A. (2006). Interaction of STIM1 with endogenously expressed human canonical TRP1 upon depletion of intracellular Ca2+ stores. *J Biol Chem, 281*(38), 28254-28264. doi:10.1074/jbc.M604272200

Ma, X., Cao, J., Luo, J., Nilius, B., Huang, Y., Ambudkar, I. S., & Yao, X. (2010). Depletion of intracellular Ca2+ stores stimulates the translocation of vanilloid transient receptor potential 4-c1 heteromeric channels to the plasma membrane. *Arterioscler Thromb Vasc Biol, 30*(11), 2249-2255. doi:10.1161/ATVBAHA.110.212084

Mangé, A., Coyaud, E., Desmetz, C., Laurent, E., Béganton, B., Coopman, P., . . . Solassol, J. (2019). FKBP4 connects mTORC2 and PI3K to activate the PDK1/Akt-dependent cell proliferation signaling in breast cancer. *Theranostics, 9*(23), 7003-7015. doi:10.7150/thno.35561

Masuda, H. a. Z. D. a. B. C. a. D. H. a. H. G. N. a. U. N. T. (2012). Role of epidermal growth factor receptor in breast cancer. In (Vol. 136, pp. 331--345).

Mayer, E. L., & Krop, I. E. (2010). Advances in targeting SRC in the treatment of breast cancer and other solid malignancies. *Clin Cancer Res, 16*(14), 3526-3532. doi:10.1158/1078-0432.CCR-09-1834

Mehta, D., Ahmmed, G. U., Paria, B. C., Holinstat, M., Voyno-Yasenetskaya, T., Tiruppathi, C., . . . Malik, A. B. (2003). RhoA interaction with inositol 1,4,5-trisphosphate receptor and transient receptor potential channel-1 regulates Ca2+ entry. Role in signaling increased endothelial permeability. *J Biol Chem, 278*(35), 33492-33500. doi:10.1074/jbc.M302401200

Miao, Y., Shen, Q., Zhang, S., Huang, H., Meng, X., Zheng, X., . . . Zou, F. (2019). Calcium-sensing stromal interaction molecule 2 upregulates nuclear factor of activated T cells 1 and transforming growth factor-β signaling to promote breast cancer metastasis. *Breast Cancer Res, 21*(1), 99. doi:10.1186/s13058-019-1185-1

Middelbeek, J., Vrenken, K., Visser, D., Lasonder, E., Koster, J., Jalink, K., . . . van Leeuwen, F. N. (2016). The TRPM7 interactome defines a cytoskeletal complex linked to neuroblastoma progression. *Eur J Cell Biol, 95*(11), 465-474. doi:10.1016/j.ejcb.2016.06.008

Moore, L. M., England, A., Ehrlich, B. E., & Rimm, D. L. (2017). Calcium Sensor, NCS-1, Promotes Tumor Aggressiveness and Predicts Patient Survival. *Mol Cancer Res, 15*(7), 942-952. doi:10.1158/1541-7786.MCR-16-0408

Moore, L. M., Wilkinson, R., Altan, M., Toki, M., Carvajal-Hausdorf, D. E., McGuire, J., . . . Rimm, D. L. (2018). An assessment of neuronal calcium sensor-1 and response to neoadjuvant chemotherapy in breast cancer patients. *NPJ Breast Cancer, 4*, 6. doi:10.1038/s41523-018-0057-7

Mound, A., Rodat-Despoix, L., Bougarn, S., Ouadid-Ahidouch, H., & Matifat, F. (2013). Molecular interaction and functional coupling between type 3 inositol 1,4,5-trisphosphate receptor and BKCa channel stimulate breast cancer cell proliferation. *Eur J Cancer, 49*(17), 3738-3751. doi:10.1016/j.ejca.2013.07.013

Mound, A., Vautrin-Glabik, A., Foulon, A., Botia, B., Hague, F., Parys, J. B., . . . Rodat-Despoix, L. (2017). Downregulation of type 3 inositol (1,4,5)-trisphosphate receptor decreases breast cancer cell migration through an oscillatory Ca. *Oncotarget, 8*(42), 72324-72341. doi:10.18632/oncotarget.20327

Nazıroğlu, M., Çiğ, B., Blum, W., Vizler, C., Buhala, A., Marton, A., . . . Pecze, L. (2017). Targeting breast cancer cells by MRS1477, a positive allosteric modulator of TRPV1 channels. *PLoS One, 12*(6), e0179950. doi:10.1371/journal.pone.0179950

Niemeyer, B. A., Bergs, C., Wissenbach, U., Flockerzi, V., & Trost, C. (2001). Competitive regulation of CaT-like-mediated Ca2+ entry by protein kinase C and calmodulin. *Proc Natl Acad Sci U S A, 98*(6), 3600-3605. doi:10.1073/pnas.051511398

Nilius, B., Prenen, J., Tang, J., Wang, C., Owsianik, G., Janssens, A., . . . Zhu, M. X. (2005). Regulation of the Ca2+ sensitivity of the nonselective cation channel TRPM4. *J Biol Chem, 280*(8), 6423-6433. doi:10.1074/jbc.M411089200

Nur, G., Nazıroğlu, M., & Deveci, H. A. (2017). Synergic prooxidant, apoptotic and TRPV1 channel activator effects of alpha-lipoic acid and cisplatin in MCF-7 breast cancer cells. *J Recept Signal Transduct Res, 37*(6), 569-577. doi:10.1080/10799893.2017.1369121

Pani, B., Ong, H. L., Brazer, S. C., Liu, X., Rauser, K., Singh, B. B., & Ambudkar, I. S. (2009). Activation of TRPC1 by STIM1 in ER-PM microdomains involves release of the channel from its scaffold caveolin-1. *Proc Natl Acad Sci U S A, 106*(47), 20087-20092. doi:10.1073/pnas.0905002106

Park, J. Y., Hwang, E. M., Yarishkin, O., Seo, J. H., Kim, E., Yoo, J., . . . Hong, S. G. (2008). TRPM4b channel suppresses store-operated Ca2+ entry by a novel protein-protein interaction with the TRPC3 channel. *Biochem Biophys Res Commun, 368*(3), 677-683. doi:10.1016/j.bbrc.2008.01.153

Peters, A. A., Milevskiy, M. J., Lee, W. C., Curry, M. C., Smart, C. E., Saunus, J. M., . . . Monteith, G. R. (2016). The calcium pump plasma membrane Ca(2+)-ATPase 2 (PMCA2) regulates breast cancer cell proliferation and sensitivity to doxorubicin. *Sci Rep, 6*, 25505. doi:10.1038/srep25505

Pillé, J. Y., Denoyelle, C., Varet, J., Bertrand, J. R., Soria, J., Opolon, P., . . . Li, H. (2005). Anti-RhoA and anti-RhoC siRNAs inhibit the proliferation and invasiveness of MDA-MB-231 breast cancer cells in vitro and in vivo. *Mol Ther, 11*(2), 267-274. doi:10.1016/j.ymthe.2004.08.029

Puram, S. V., Riccio, A., Koirala, S., Ikeuchi, Y., Kim, A. H., Corfas, G., & Bonni, A. (2011). A TRPC5-regulated calcium signaling pathway controls dendrite patterning in the mammalian brain. *Genes Dev, 25*(24), 2659-2673. doi:10.1101/gad.174060.111

Redondo, P. C., Jardin, I., Lopez, J. J., Salido, G. M., & Rosado, J. A. (2008). Intracellular Ca2+ store depletion induces the formation of macromolecular complexes involving hTRPC1, hTRPC6, the type II IP3 receptor and SERCA3 in human platelets. *Biochim Biophys Acta, 1783*(6), 1163-1176. doi:10.1016/j.bbamcr.2007.12.008

Rennstam, K., McMichael, N., Berglund, P., Honeth, G., Hegardt, C., Rydén, L., . . . Hedenfalk, I. (2010). Numb protein expression correlates with a basal-like phenotype and cancer stem cell markers in primary breast cancer. *Breast Cancer Res Treat, 122*(2), 315-324. doi:10.1007/s10549-009-0568-x

Rivas, J., Díaz, N., Silva, I., Morales, D., Lavanderos, B., Álvarez, A., . . . Cerda, O. (2020a). KCTD5, a novel TRPM4-regulatory protein required for cell migration as a new predictor for breast cancer prognosis. *FASEB J*. doi:10.1096/fj.201901195RRR

Rivas, J., Díaz, N., Silva, I., Morales, D., Lavanderos, B., Álvarez, A., . . . Cerda, O. (2020b). KCTD5, a novel TRPM4-regulatory protein required for cell migration as a new predictor for breast cancer prognosis. *FASEB J, 34*(6), 7847-7865. doi:10.1096/fj.201901195RRR

Rosado, J. A., & Sage, S. O. (2001). Activation of store-mediated calcium entry by secretion-like coupling between the inositol 1,4,5-trisphosphate receptor type II and human transient receptor potential (hTrp1) channels in human platelets. *Biochem J, 356*(Pt 1), 191-198. doi:10.1042/0264-6021:3560191

Rutter, A. R., Ma, Q. P., Leveridge, M., & Bonnert, T. P. (2005). Heteromerization and colocalization of TrpV1 and TrpV2 in mammalian cell lines and rat dorsal root ganglia. *Neuroreport, 16*(16), 1735-1739. doi:10.1097/01.wnr.0000185958.03841.0f

Salas, M. M., Hargreaves, K. M., & Akopian, A. N. (2009). TRPA1-mediated responses in trigeminal sensory neurons: interaction between TRPA1 and TRPV1. *Eur J Neurosci, 29*(8), 1568-1578. doi:10.1111/j.1460-9568.2009.06702.x

Schnelzer, A., Prechtel, D., Knaus, U., Dehne, K., Gerhard, M., Graeff, H., . . . Lengyel, E. (2000). Rac1 in human breast cancer: overexpression, mutation analysis, and characterization of a new isoform, Rac1b. *Oncogene, 19*(26), 3013-3020. doi:10.1038/sj.onc.1203621

Schoeber, J. P., Topala, C. N., Wang, X., Diepens, R. J., Lambers, T. T., Hoenderop, J. G., & Bindels, R. J. (2006). RGS2 inhibits the epithelial Ca2+ channel TRPV6. *J Biol Chem, 281*(40), 29669-29674. doi:10.1074/jbc.M606233200

Sehgal, P., Szalai, P., Olesen, C., Praetorius, H. A., Nissen, P., Christensen, S. B., . . . Møller, J. V. (2017). Inhibition of the sarco/endoplasmic reticulum (ER) Ca^2+^-ATPase by thapsigargin analogs induces cell death via ER Ca2+ depletion and the unfolded protein response. *J Biol Chem, 292*(48), 19656-19673. doi:10.1074/jbc.M117.796920

Shi, J., Ju, M., Saleh, S. N., Albert, A. P., & Large, W. A. (2010). TRPC6 channels stimulated by angiotensin II are inhibited by TRPC1/C5 channel activity through a Ca2+- and PKC-dependent mechanism in native vascular myocytes. *J Physiol, 588*(Pt 19), 3671-3682. doi:10.1113/jphysiol.2010.194621

Singh, B. B., Liu, X., Tang, J., Zhu, M. X., & Ambudkar, I. S. (2002). Calmodulin Regulates Ca2+-Dependent Feedback Inhibition of Store-Operated Ca2+ Influx by Interaction with a Site in the C Terminus of TrpC1. *Molecular Cell, 9*(4), 739 - 750. doi:https://doi.org/10.1016/S1097-2765(02)00506-3

Sinkins, W. G., Goel, M., Estacion, M., & Schilling, W. P. (2004). Association of immunophilins with mammalian TRPC channels. *J Biol Chem, 279*(33), 34521-34529. doi:10.1074/jbc.M401156200

Sokabe, T., Fukumi-Tominaga, T., Yonemura, S., Mizuno, A., & Tominaga, M. (2010). The TRPV4 channel contributes to intercellular junction formation in keratinocytes. *J Biol Chem, 285*(24), 18749-18758. doi:10.1074/jbc.M110.103606

Sternfeld, L., Anderie, I., Schmid, A., Al-Shaldi, H., Krause, E., Magg, T., . . . Schulz, I. (2007). Identification of tyrosines in the putative regulatory site of the Ca2+ channel TRPV6. *Cell Calcium, 42*(1), 91-102. doi:10.1016/j.ceca.2006.11.008

Stokes, A. J., Shimoda, L. M., Koblan-Huberson, M., Adra, C. N., & Turner, H. (2004). A TRPV2-PKA signaling module for transduction of physical stimuli in mast cells. *J Exp Med, 200*(2), 137-147. doi:10.1084/jem.20032082

Strübing, C., Krapivinsky, G., Krapivinsky, L., & Clapham, D. E. (2001). TRPC1 and TRPC5 form a novel cation channel in mammalian brain. *Neuron, 29*(3), 645-655. doi:10.1016/s0896-6273(01)00240-9

Stumpf, T., Zhang, Q., Hirnet, D., Lewandrowski, U., Sickmann, A., Wissenbach, U., . . . Fecher-Trost, C. (2008). The human TRPV6 channel protein is associated with cyclophilin B in human placenta. *J Biol Chem, 283*(26), 18086-18098. doi:10.1074/jbc.M801821200

Takahashi, N., Chen, H. Y., Harris, I. S., Stover, D. G., Selfors, L. M., Bronson, R. T., . . . Brugge, J. S. (2018). Cancer Cells Co-opt the Neuronal Redox-Sensing Channel TRPA1 to Promote Oxidative-Stress Tolerance. *Cancer Cell, 33*(6), 985-1003.e1007. doi:10.1016/j.ccell.2018.05.001

Takayama, Y., Shibasaki, K., Suzuki, Y., Yamanaka, A., & Tominaga, M. (2014). Modulation of water efflux through functional interaction between TRPV4 and TMEM16A/anoctamin 1. *FASEB J, 28*(5), 2238-2248. doi:10.1096/fj.13-243436

Tang, J., Lin, Y., Zhang, Z., Tikunova, S., Birnbaumer, L., & Zhu, M. X. (2001). Identification of common binding sites for calmodulin and inositol 1,4,5-trisphosphate receptors on the carboxyl termini of trp channels. *J Biol Chem, 276*(24), 21303-21310. doi:10.1074/jbc.M102316200

Tian, D., Jacobo, S. M., Billing, D., Rozkalne, A., Gage, S. D., Anagnostou, T., . . . Greka, A. (2010). Antagonistic regulation of actin dynamics and cell motility by TRPC5 and TRPC6 channels. *Sci Signal, 3*(145), ra77. doi:10.1126/scisignal.2001200

Tomiguchi, M., Yamamoto, Y., Yamamoto-Ibusuki, M., Goto-Yamaguchi, L., Fujiki, Y., Fujiwara, S., . . . Iwase, H. (2016). Fibroblast growth factor receptor-1 protein expression is associated with prognosis in estrogen receptor-positive/human epidermal growth factor receptor-2-negative primary breast cancer. *Cancer Sci, 107*(4), 491-498. doi:10.1111/cas.12897

Tu, C. L., Chang, W., & Bikle, D. D. (2005). Phospholipase cgamma1 is required for activation of store-operated channels in human keratinocytes. *J Invest Dermatol, 124*(1), 187-197. doi:10.1111/j.0022-202X.2004.23544.x

Uhlen, M., Zhang, C., Lee, S., Sjöstedt, E., Fagerberg, L., Bidkhori, G., . . . Ponten, F. (2017). A pathology atlas of the human cancer transcriptome. *Science, 357*(6352). doi:10.1126/science.aan2507

van de Graaf, S. F., Hoenderop, J. G., & Bindels, R. J. (2006). Regulation of TRPV5 and TRPV6 by associated proteins. *Am J Physiol Renal Physiol, 290*(6), F1295-1302. doi:10.1152/ajprenal.00443.2005

Vautrin-Glabik, A., Botia, B., Kischel, P., Ouadid-Ahidouch, H., & Rodat-Despoix, L. (2018). IP_3_ R3 silencing induced actin cytoskeletal reorganization through ARHGAP18/RhoA/mDia1/FAK pathway in breast cancer cell lines. *Biochim Biophys Acta Mol Cell Res, 1865*(7), 945-958. doi:10.1016/j.bbamcr.2018.04.002

Vinuela-Fernandez, I., Sun, L., Jerina, H., Curtis, J., Allchorne, A., Gooding, H., . . . Fleetwood-Walker, S. (2014). The TRPM8 channel forms a complex with the 5-HT(1B) receptor and phospholipase D that amplifies its reversal of pain hypersensitivity. *Neuropharmacology, 79*, 136-151. doi:10.1016/j.neuropharm.2013.11.006

Wang, H., Zou, L., Ma, K., Yu, J., Wu, H., Wei, M., & Xiao, Q. (2017). Cell-specific mechanisms of TMEM16A Ca^2+^-activated chloride channel in cancer. *Mol Cancer, 16*(1), 152. doi:10.1186/s12943-017-0720-x

Wang, M. Y., Huang, H. Y., Kuo, Y. L., Lo, C., Sun, H. Y., Lyu, Y. J., . . . Chen, P. S. (2019). TARBP2-Enhanced Resistance during Tamoxifen Treatment in Breast Cancer. *Cancers (Basel), 11*(2). doi:10.3390/cancers11020210

Wang, S., Wang, N., Zheng, Y., Yang, B., Liu, P., Zhang, F., . . . Wang, Z. (2020). Caveolin-1 inhibits breast cancer stem cells via c-Myc-mediated metabolic reprogramming. *Cell Death Dis, 11*(6), 450. doi:10.1038/s41419-020-2667-x

Wang, Y., Qi, Y. X., Qi, Z., & Tsang, S. Y. (2019). TRPC3 Regulates the Proliferation and Apoptosis Resistance of Triple Negative Breast Cancer Cells through the TRPC3/RASA4/MAPK Pathway. *Cancers (Basel), 11*(4). doi:10.3390/cancers11040558

Wang, Y., Zhou, X., Zhu, H., Liu, S., Zhou, C., Zhang, G., . . . Xu, N. (2005). Overexpression of EB1 in human esophageal squamous cell carcinoma (ESCC) may promote cellular growth by activating beta-catenin/TCF pathway. *Oncogene, 24*(44), 6637-6645. doi:10.1038/sj.onc.1208819

Weber, L. V., Al-Refae, K., Wölk, G., Bonatz, G., Altmüller, J., Becker, C., . . . Hatt, H. (2016). Expression and functionality of TRPV1 in breast cancer cells. *Breast Cancer (Dove Med Press), 8*, 243-252. doi:10.2147/BCTT.S121610

Woo, S. K., Kwon, M. S., Ivanov, A., Gerzanich, V., & Simard, J. M. (2013). The sulfonylurea receptor 1 (Sur1)-transient receptor potential melastatin 4 (Trpm4) channel. *J Biol Chem, 288*(5), 3655-3667. doi:10.1074/jbc.M112.428219

Xie, F., Jin, K., Shao, L., Fan, Y., Tu, Y., Li, Y., . . . Zhang, L. (2017). FAF1 phosphorylation by AKT accumulates TGF-β type II receptor and drives breast cancer metastasis. *Nat Commun, 8*, 15021. doi:10.1038/ncomms15021

Xie, Y. G., Yu, Y., Hou, L. K., Wang, X., Zhang, B., & Cao, X. C. (2016). FYN promotes breast cancer progression through epithelial-mesenchymal transition. *Oncol Rep, 36*(2), 1000-1006. doi:10.3892/or.2016.4894

Xiong, H., Chen, Z., Zheng, W., Sun, J., Fu, Q., Teng, R., . . . Zhou, J. (2020). FKBP4 is a malignant indicator in luminal A subtype of breast cancer. *J Cancer, 11*(7), 1727-1736. doi:10.7150/jca.40982

Xu, H., Zhao, H., Tian, W., Yoshida, K., Roullet, J. B., & Cohen, D. M. (2003). Regulation of a transient receptor potential (TRP) channel by tyrosine phosphorylation. SRC family kinase-dependent tyrosine phosphorylation of TRPV4 on TYR-253 mediates its response to hypotonic stress. *J Biol Chem, 278*(13), 11520-11527. doi:10.1074/jbc.M211061200

Xu, L., Zhang, Y., Qu, X., Che, X., Guo, T., Cai, Y., . . . Liu, Y. (2017). E3 Ubiquitin Ligase Cbl-b Prevents Tumor Metastasis by Maintaining the Epithelial Phenotype in Multiple Drug-Resistant Gastric and Breast Cancer Cells. *Neoplasia, 19*(4), 374-382. doi:10.1016/j.neo.2017.01.011

Yang, S., Zhang, J. J., & Huang, X. Y. (2009). Orai1 and STIM1 are critical for breast tumor cell migration and metastasis. *Cancer Cell, 15*(2), 124-134. doi:10.1016/j.ccr.2008.12.019

Yoon, H. J., Kim, D. H., Kim, S. J., Jang, J. H., & Surh, Y. J. (2019). Src-mediated phosphorylation, ubiquitination and degradation of Caveolin-1 promotes breast cancer cell stemness. *Cancer Lett, 449*, 8-19. doi:10.1016/j.canlet.2019.01.021

Yuan, J. P., Kiselyov, K., Shin, D. M., Chen, J., Shcheynikov, N., Kang, S. H., . . . Worley, P. F. (2003). Homer binds TRPC family channels and is required for gating of TRPC1 by IP3 receptors. *Cell, 114*(6), 777-789. doi:10.1016/s0092-8674(03)00716-5

Yuan, J. P., Zeng, W., Huang, G. N., Worley, P. F., & Muallem, S. (2007). STIM1 heteromultimerizes TRPC channels to determine their function as store-operated channels. *Nat Cell Biol, 9*(6), 636-645. doi:10.1038/ncb1590

Zhao, Y., Wang, J., Jiang, H., Yu, Z., Li, X., & Shi, J. (2015). Following OGD/R, annexin 1 nuclear translocation and subsequent induction of apoptosis in neurons are assisted by myosin IIA in a TRPM7 kinase-dependent manner. *Mol Neurobiol, 51*(2), 729-742. doi:10.1007/s12035-014-8781-y

Zhu, Z., Jiao, L., Li, T., Wang, H., Wei, W., & Qian, H. (2018). Expression of AQP3 and AQP5 as a prognostic marker in triple-negative breast cancer. *Oncol Lett, 16*(2), 2661-2667. doi:10.3892/ol.2018.8955

Zimmermann, J., Latta, L., Beck, A., Leidinger, P., Fecher-Trost, C., Schlenstedt, G., . . . Flockerzi, V. (2014). Trans-activation response (TAR) RNA-binding protein 2 is a novel modulator of transient receptor potential canonical 4 (TRPC4) protein. *J Biol Chem, 289*(14), 9766-9780. doi:10.1074/jbc.M114.557066
